# Barriers to accessing healthcare for people with disabilities:a systematic review

**DOI:** 10.3389/fpubh.2026.1765145

**Published:** 2026-02-19

**Authors:** M. Chimienti, G. Morlino, D. Ndreca, K. Stefanidhi, O. Zerellari, R. Shkreli, S. Gjergji, F. Leonforte, V. Nicosia, A. Mistretta, E. Buonomo, G. Liotta, L. Palombi

**Affiliations:** 1 Catholic University Our Lady of Good Counsel, Tirana, Albania; 2 Department of Biomedicine and Prevention, University of Rome Tor Vergata, Rome, Italy; 3 Department of Integrated Hygiene, Organizational and Service Activities (Structural Department), Health Management, University Hospital Polyclinic “G. Rodolico-San Marco”, Catania, Italy; 4 Department of Medical and Surgical Sciences and Advanced Technologies “G.F Ingrassia”, University of Catania, Catania, Italy

**Keywords:** access, barriers, disability, health services, inequalities, quality of Life

## Abstract

**Objective:**

This review identifies global evidence on barriers to healthcare access for disabled persons and healthcare service efficiency. It highlights recurring patterns, systemic discrimination, and evidence gaps. The review supports policy development, health system redesign, and future research to advance disability-inclusive health equity.

**Methods:**

Strict inclusion and exclusion criteria were applied. The investigation used PubMed, Scopus, and Web of Science with rigorous double-blind screening and analysis.

**Results:**

Of 6,570 articles, 86 studies were selected (28 mental/cognitive/developmental, 33 physical, 25 multiple disabilities) including diverse adult age groups. Barriers included physical, financial, transportation, communication, attitudinal, organizational, knowledge-related, sensory, cultural, and systemic hurdles.

Critical gaps include disproportionate focus on barriers rather than interventions (only 10%), implicit facilitators, underrepresentation of stakeholder perspectives, and geographic concentration in high-income countries.

**Conclusions:**

Significant barriers hinder service access. Most frequent were financial constraints, transportation challenges, communication difficulties, limited physical accessibility, provider knowledge gaps, and socio-cultural stigma. Accessibility is typically addressed through individualized accommodations rather than systematic universal design. Providers frequently lack training and hold negative attitudes; technology retrofits rather than design inclusively. Effective solutions require systemic redesign and placing disability at the center of health equity efforts.

## Introduction

Individuals with disabilities are defined as those who possess physical, mental, intellectual, or sensory impairments that, due to interaction with various barriers, hinder their full participation in society (1). The concept of disability has evolved from a solely illness-based, corrective medical approach to a more integrated view, recognizing disability as the result of personal, environmental, and social factors (2). They represent approximately 15% of the global population affecting more than 1 billion people (3). In 2019, one in every eight people, or 970 million people around the world were living with a mental disorder (4). Specifically, physical disability rates are often reported as around 1.4% in high-income countries and can be higher in low- and middle-income countries (5). These disparities contribute to avoidable suffering, reduced quality of life, and preventable premature mortality, making healthcare accessibility a public health priority.

An essential right and a critical aspect to a person's well being is access to healthcare and social-health services. This analysis demonstrates that while everyone should enjoy access to healthcare adapted to their needs without discrimination, this goal remains unmet in many parts of the world (6). Some researchers have identified numerous barriers including financial and economic, physical access and transportation, provider knowledge and training barriers and system organization and coordination barriers. Financial barriers, such as limited insurance coverage, cost of specialized services or socioeconomic disparities are one of the invisible walls patients encounter in their journey. Physical barriers, including transportation challenges, lack of trained staff, inaccessible healthcare facilities but also inaccessible housing near healthcare providers are considered to be major structural obstacles. Additionally, lack of accessible communication and digital accessibility issues have a huge impact on their quality of life discouraging them from seeking care.

While numerous studies have documented challenges to healthcare access, the evidence remains fragmented across disability types and health systems, context-specific, and descriptive. Much of the research focuses on individual-level barriers without integrating them into a broader view. Furthermore, limited research evaluates the effectiveness of interventions and also the perspectives of people with disabilities leaving a critical gap in knowledge about how healthcare systems can be redesigned to achieve disability-inclusive equity. Recent reviews confirm disabled individuals face barriers including transportation difficulties, physical inaccessibility, and provider knowledge gaps, with rural populations experiencing 13–40% higher unmet needs, while legal frameworks remain insufficient without multi-sector collaborative partnerships (7, 8).

This systematic review aims to identify, map and synthesize global evidence on barriers to healthcare access for people with disabilities and how effective is the access to healthcare and social- health services. Specifically, it seeks to identify recurring patterns, highlight systemic and structural dimensions of discrimination, and reveal areas where evidence remains limited. By doing so, this review provides a foundation for policy development, health system redesign, and future research to advance disability-inclusive health equity.

## Materials and methods

### Eligibility criteria

The PECO(S) framework was used for this systematic review; the criteria used are shown in Table 1.

**Table 1:** PECO(S) Framework.

**PECOS**	**Inclusion criteria**	**Exclusion criteria**
(P)opulation	- People with various disabilities (physical, sensory, mental, cognitive, and developmental, communicative)“. - >18 years old	- People without disabilities - < 18 years
(E)xposition	- Social and health care needs for access to medical treatment	- Studies not analyzing interventions, care models and service approaches
(C)omparator	- People without disabilities (where available). - >18 years old	
(O)utcome	- Identification of barriers, the degree of access to services, and their impact on life (comorbidity, mortality, and morbidity)	- Studies that do not value barriers to access to healthcare and social care for people with disabilities and their impact on quality of life
(S)tudy design	- Case reports; - Case series; - Cross sectional studies; - Cohort studies; - Case control studies; - Qualitative studies; - Quasi experimental studies; - RCT (randomized clinical trials); - Mixed methods studies	- Book chapter; - Editorial; - Commentary; - Letter to the editor; - Review
Other criteria	- Studies published after 2014; - Included both quantitative and qualitative data	- Not written in English; - Full text not available; - Studies published before 2014

### Research methods

The systematic review was conducted in accordance with the international PRISMA (9) guidelines, with the aim of mapping the available evidence on barriers to accessing healthcare and social care services for people with disabilities. Based on the research questions, three search strings were developed to identify relevant studies in the following electronic databases: PubMed, Scopus, and Web of Science.

Specific search strings combining terms related to access to care were adapted for each database in order to optimize information retrieval.

The search identified studies on healthcare access barriers and facilitators for adults with disabilities from 2014 onwards. The 2014 cutoff captures post-CRPD implementation evidence and contemporary healthcare systems while ensuring sufficient evidence volume. The search combined three concept groups: disability terms (disability, impairment, physical, sensory, mental, cognitive, and developmental, communicative disability), healthcare access terms (access, barriers, facilitators, utilization), and outcomes (quality of life, equity, disparities). Database-specific controlled vocabulary (MeSH- Medical Subject Headings- for PubMed) was combined with free-text keywords to maximize sensitivity while maintaining specificity.

Below are the strings for PubMed, Scopus, and Web of Science, respectively (Table 2).

**Table 2:** Search strings.

**•PubMed**
(“access to healthcare” OR “barriers” OR “challenges” OR “disparities” OR “inequalities” OR “limitations”) AND (‘disability' OR “people with disabilities” OR “physical impairment” OR “mental disabilities” OR “chronic diseases” OR “cognitive disabilities” OR “developmental disabilities”) AND (“health services” OR “medical care” OR “treatment” OR “support services” OR “rehabilitation services” OR “primary care” OR “social services” OR “telemedicine” OR “community health care”) AND (“quality of life” OR “health impact” OR “patient outcomes” OR “morbidity” OR “mortality” OR “comorbidity”)
•**Scopus**
(“access to healthcare” OR “access to healthcare” OR “barriers” OR “challenges” OR “disparities” OR “inequalities” OR ‘limitations') AND (“disabil^*^” OR “handicap” OR “impairment” OR “functional limitations”) AND (“health care” OR “health services” OR “medical care”) AND (“quality of life” OR “health outcomes” OR “well-being” OR ‘morbidity' OR “mortality”)
•**Web of science**
TS=(“access to healthcare” OR “access to healthcare” OR “barriers” OR “challenges” OR “disparities” OR ‘inequalities' OR ‘limitations') AND (“disabil^*^” OR “handicap” OR “impairment” OR “functional limitations”) AND (“health care” OR “health services” OR “medical care”) AND (“quality of life” OR “health outcomes” OR “well-being” OR ‘morbidity' OR “mortality”)

### Study selection and data extraction

Two independent reviewers selected the studies in two stages: titles and abstract screening to assess relevance, followed by full-text review of the articles selected in the first stage to verify eligibility based on the inclusion and exclusion criteria. Any disagreements were resolved through discussion.

Relevant data were extracted from the included studies using a predefined grid, to extract information on: study characteristics (author, year, country, study design, objectives, sample size, type of care, type of health service, data collection methods, data analysis methods), participant characteristics (type of disability, age, gender, experiences of individuals with disabilities and their caregivers), barriers and facilitators to access, and relevant outcomes.

The data extraction grid was developed iteratively by the research team. Categories were selected based on: PRISMA standards and the PECO(S) framework; disability-specific barriers identified in preliminary scoping searches (financial, transportation, communication, provider knowledge); and stakeholder perspectives.

Caregiver experiences were included as a distinct category because caregivers often serve as healthcare navigators and advocates for people with disabilities, and their burden directly impacts care access. The grid was pilot-tested on five diverse articles (varying by disability type and study design), refined through team discussion, and finalized by consensus.

### Evaluation of study quality

The evaluation of study quality was conducted using a variety of scales, using multiple validated appraisal tools, selected based on established best practices for systematic reviews incorporating diverse study designs. Three distinct checklists were utilized in the course of the study, with the CASP (Critical Appraisal Skills Programme) checklist (10) was used for the majority of studies, including cross-sectional, cohort, and qualitative studies, as well as RCTs, because it provides design-specific checklists with clear quality criteria applicable across these methodologies. The JBI (Joanna Briggs Institute) (11) checklist was utilized for case report studies due to its specialized criteria for evaluating case-based evidence, while the MMAT (Mixed Methods Appraisal Tool) (12) was employed for mixed methods studies as it is specifically designed to assess both qualitative and quantitative components within a single study, which cannot be adequately evaluated using single-method tools. This approach follows recommendations for systematic reviews including heterogeneous evidence, where different study designs require tailored quality assessment criteria to avoid inappropriate application of quality standards across incompatible methodologies.

Two reviewers assessed each study independently, resolving any discrepancies through discussion or consultation with a senior reviewer. Scores were converted into percentages to facilitate quality assessment.

## Results

The systematic analysis of the literature led to the identification of 6,570 articles: 2,740 from PubMed, 1,347 from Scopus, and 2,483 from Web of Science.

**Figure 1:**
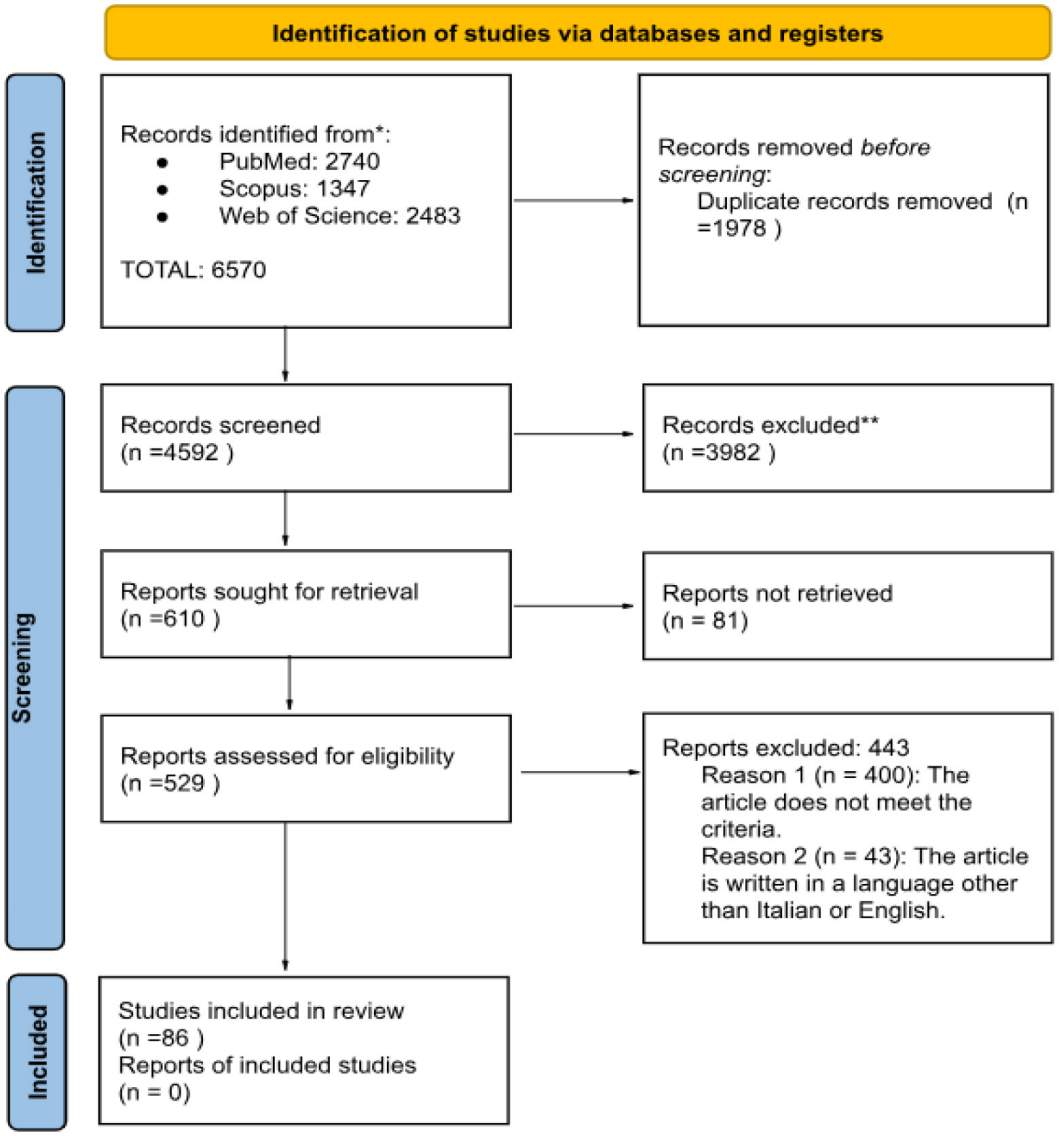
Flowchart diagram.

After excluding 1,978 duplicates, the remaining documents were selected based on their titles and abstracts. Of the 529 articles included, a full-text reading excluded 443 articles, 43 of which were excluded because they were not in English or Italian, and 400 because they did not meet the Criteria. Finally, our selection led to the identification of a total of 86 documents included in the review. The article selection process is shown in the flow chart (Figure 1).

### Risk of bias

**Figure 2:**
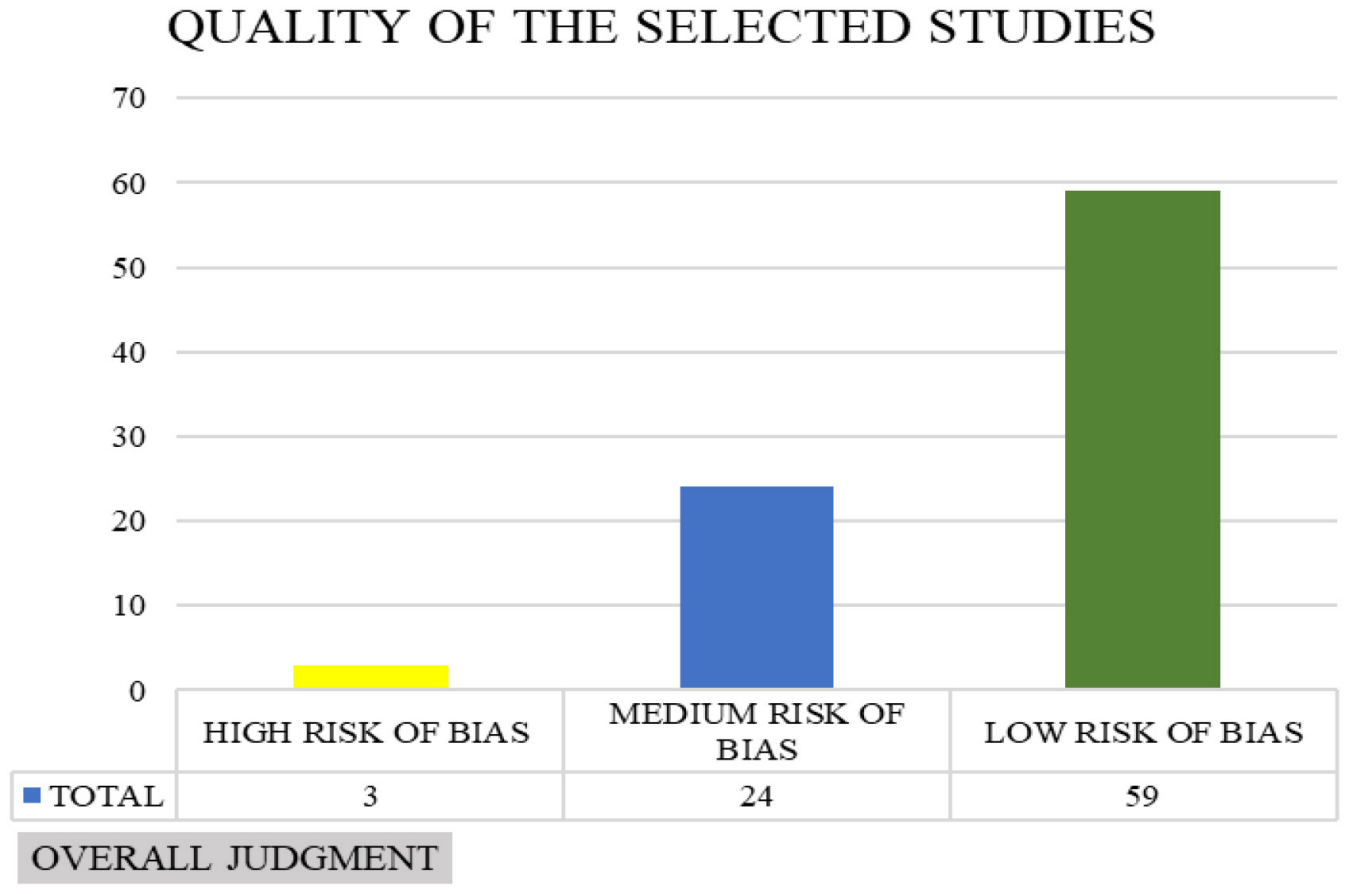
Overall judgement of risk of bias evaluation of selected articles.

In assessing the risk of bias in qualitative, RCT, observational and mixed-method articles, scales appropriate to the type of study were used, accepting only two error notes to define the article as high quality: as can be seen in Figure 2, the number of articles with a low risk of bias is 59, the number of articles with a medium risk of bias is 24 and the number of articles with a high risk of bias is three. The summary tables of the bias risk assessment of the selected articles are presented in the Supplementary material (S4-S9).

Quality assessment informed interpretation but not inclusion decisions. All eligible studies were retained to maximize evidence mapping across diverse contexts and populations. During synthesis, the research team considered the quality of supporting evidence when drawing conclusions, with awareness that the majority of findings were derived from low risk-of-bias studies, providing confidence in the robustness of identified patterns.

### Data extraction of selected studies

The complete extraction tables can be found in the Supplementary Tables S1, S2.

### Narrative synthesis

### Study characteristics

A total of 86 articles were included in this review. Geographically, 30 studies were conducted in North America (United States and Canada), 18 in Europe (UK, Germany, Ireland, Poland), 13 in Asia (Afghanistan, China, South Korea, India, Lao PDR, Saudi Arabia, Iran, Nepal, Philippines), 12 in Africa (South Africa, Ghana, Ethiopia, Uganda, Malawi, Nigeria, Sub-Saharan Africa), 9 in Oceania (Australia and New Zealand), and one in South America (Brazil). The most represented countries were the United States with 25 articles (13–37), UK with 13 articles (38–50), Australia with 7 (51–57), South Africa with 5 (58–62) and Canada with 5 (63–67). In addition, three studies were classified as international (68–70).

The publication years ranged from 2014 to 2025.

Regarding the methodological approach, 52 studies employed quantitative methods, 29 utilized qualitative methods, and five used mixed-methods.

Among quantitative studies, the most common study design was cross sectional including 36 articles (14, 15, 17–21, 24, 25, 28–34, 38, 46, 51, 53, 58, 61, 68, 70–82), followed by 15 Cohort studies (22, 35, 36, 39, 43, 45, 56, 57, 62–64, 69, 83–85) and 1 RCT (randomized clinical trial) (16). Data for these studies were primarily collected via surveys or analysis of administrative records.

The qualitative studies include (13, 23, 26, 27, 37, 40–42, 48–50, 52, 54, 59, 65–67, 86–96) and 1 case report study (44) largely adopted descriptive or phenomenology designs, primarily using semi-structured interviews and focus groups for data collection.

Finally, the 5 mixed-methods studies (47, 55, 60, 97, 98) combined surveys with in-depth interviews.

By disability category, 33 studies focused on physical disabilities (21–27, 30, 32, 33, 35, 37, 48–50, 55, 56, 58, 59, 61, 62, 66, 69, 70, 76–78, 89–91, 93, 94, 96), 28 on mental, cognitive, and developmental disabilities category (13–18, 38–46, 51, 53, 54, 57, 63, 67, 71–73, 86, 87, 97, 98), including psychiatric conditions (e.g., schizophrenia, depression), cognitive impairments (e.g., dementia), and developmental disabilities (e.g., autism, intellectual disabilities), and 25 addressed multiple disabilities (19, 20, 28, 29, 31, 34, 36, 47, 52, 60, 64, 65, 68, 74, 75, 79–85, 88, 92, 95).

The majority of studies examined general adult populations (≥18 years), with a trend toward middle-aged adults. Gender representation was reported in 75 studies as mixed, seven were unspecified, six focused exclusively on females, and 1 on males. Sample sizes ranged widely from 1 participant (44) to 35,773,286 (15).

Regarding healthcare system classification, 48 studies were conducted in universal systems, 36 in mixed systems, one in private systems, and one did not specify.

Across the studies, the types of care examined ranged from multidisciplinary in 25 articles (14, 20, 24, 30, 32–35, 42, 47, 52, 55, 56, 60, 65, 70, 75–77, 80, 82, 86, 89, 97, 98) to primary healthcare services in 19 articles (15, 16, 18–21, 29, 37–39, 41, 46, 51, 63, 67, 73, 88, 92, 95), rehabilitation services, general healthcare, hospital care, specialist services, preventive care and community-based services.

Data collection methods varied, with surveys being the most common, followed by interviews, administrative data and medical records, secondary data analysis, literature reviews, and mixed approaches.

The main objectives across the studies were diverse, including the identification of barriers and challenges to healthcare access, the examination of healthcare disparities and inequalities, the assessment of disease prevalence and health outcomes, the evaluation of healthcare service delivery and models, the exploration of patient and provider experiences, and the analysis of rehabilitation and therapy services. Additional objectives focused on interventions and programs, access to healthcare in general, the role of digital health and technology, as well as preventive care and screening, alongside other mixed aims.

Regarding outcomes, an analysis of our studies reveals ~30% analyzed disease burden outcomes related to more chronic conditions occurring simultaneously, higher mortality rates and more severe morbidity when conditions do occur (14, 15, 17, 21, 22, 29, 32, 43–45, 53, 56, 58, 61–63, 71, 74, 83–85, 89, 98).

### Barriers to accessing the healthcare system

**Figure 3:**
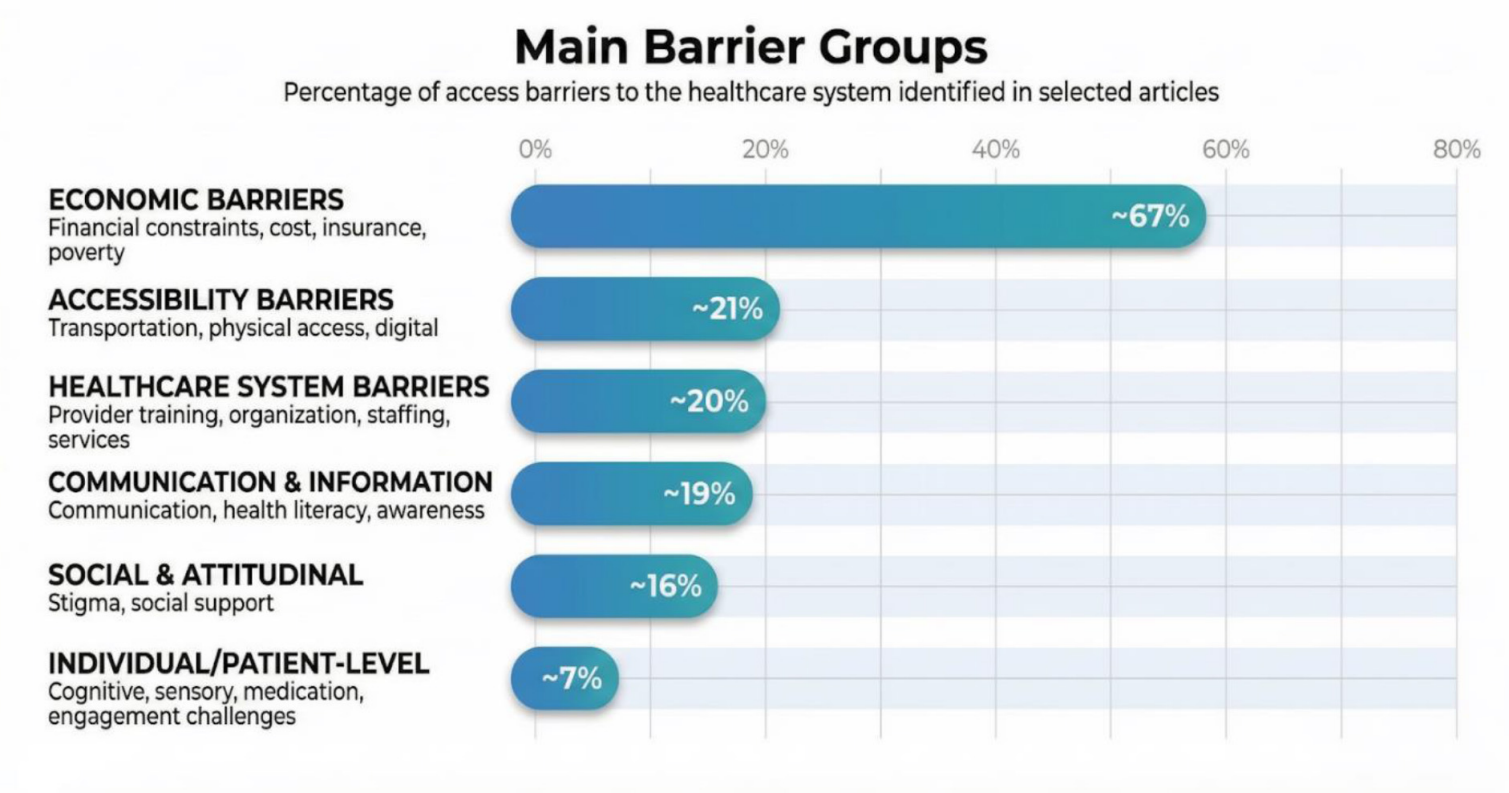
Mapping of barriers to access in selected articles.

Commonly reported barriers included financial and economic constraints (15, 19, 20, 25, 26, 32, 34, 58, 61, 65, 66, 70, 74, 80–82, 89–92, 95), physical access and transportation difficulties (19, 24–26, 30, 33, 48, 52, 54, 59–61, 74, 75, 79, 88–90, 92, 95), followed by inadequate provider knowledge and training, and system-level issues related to organization and coordination as shown on Figure 3. Reported facilitators of access included system-level reforms, technology integration, person-centered approaches, and family or caregiver support.

Barriers and facilitators were analyzed across three primary categories of disability. A significant proportion of the articles identified multiple barriers.

In the context of mental, cognitive, and developmental disabilities, the most prevalent type is communication and cognitive barriers, which is represented in 89% of the studies (13–15, 17, 18, 38–46, 51, 53, 54, 57, 63, 67, 71, 73, 86, 97, 98). This is followed by healthcare provider knowledge and training gaps, which is represented in 75% (13, 14, 17, 18, 38, 39, 41–46, 51, 53, 54, 57, 63, 67, 71, 73, 86, 87, 97, 98) of the studies, system & service coordination barriers in 68% (14, 16, 17, 39, 41–45, 53, 54, 57, 63, 67, 71, 73, 87, 97, 98) and financial & economic barriers in 54% (13–17, 40, 51, 54, 57, 63, 72, 73, 87, 97, 98). In a smaller percentage are diagnostic & screening barriers in 43% and sensory & environmental barriers in 36% of the studies. The percentages indicate the share of studies that reported barriers within each category.

In the context of research pertaining to multiple disabilities, 96% of studies noted the presence of financial and economic barriers (19, 20, 28, 29, 31, 34, 47, 52, 60, 64, 65, 68, 74, 75, 79–85, 88, 92, 95), 68% identified issues related to inaccessible transportation, long travel distances, rural residence, lack of paved roads, remote locations (19, 20, 28, 29, 31, 52, 60, 64, 65, 74, 75, 79, 80, 85, 88, 92, 95) and 64% reported barriers related to physical accessibility and infrastructure (19, 20, 28, 29, 60, 64, 65, 75, 79, 80, 82, 83, 85, 88, 92, 95).

In the context of physical disability, economic and financial barriers emerged as the most prevalent barriers (25, 26, 30, 32, 33, 35, 37, 48, 50, 55, 56, 59, 61, 66, 69, 70, 76–78, 89–91, 94, 96), accounting for 82% of cases. This was followed by transportation difficulties in 64% (24–26, 30, 32, 33, 48, 59, 61, 69, 70, 76–78, 89–91, 93, 96) and healthcare provider and system issues (60%) (22, 23, 26, 37, 48–50, 55, 56, 58, 59, 69, 70, 77, 89–91, 94, 96).

Lived experiences varied across contexts. Many individuals reported difficulties accessing healthcare services, barriers to quality care, mental health and psychological consequences, physical and environmental challenges, communication and information gaps, stigma and discrimination, economic barriers, employment and independence concerns, and challenges in managing specific medical conditions. Ten studies did not report on lived experiences (22, 25, 29, 31, 35, 39, 42, 67, 81, 84).

Caregiver perspectives on the other hand were largely absent, but in those reported the most common experiences were physical and daily care burden, financial and economic impact, system coordination and management, communication and information challenges, social and family relationships, healthcare navigation and advocacy, emotional and psychological impact, training and education needs, transportation and access barriers and professional work impact.

### Facilitators of access to the healthcare system

**Figure 4:**
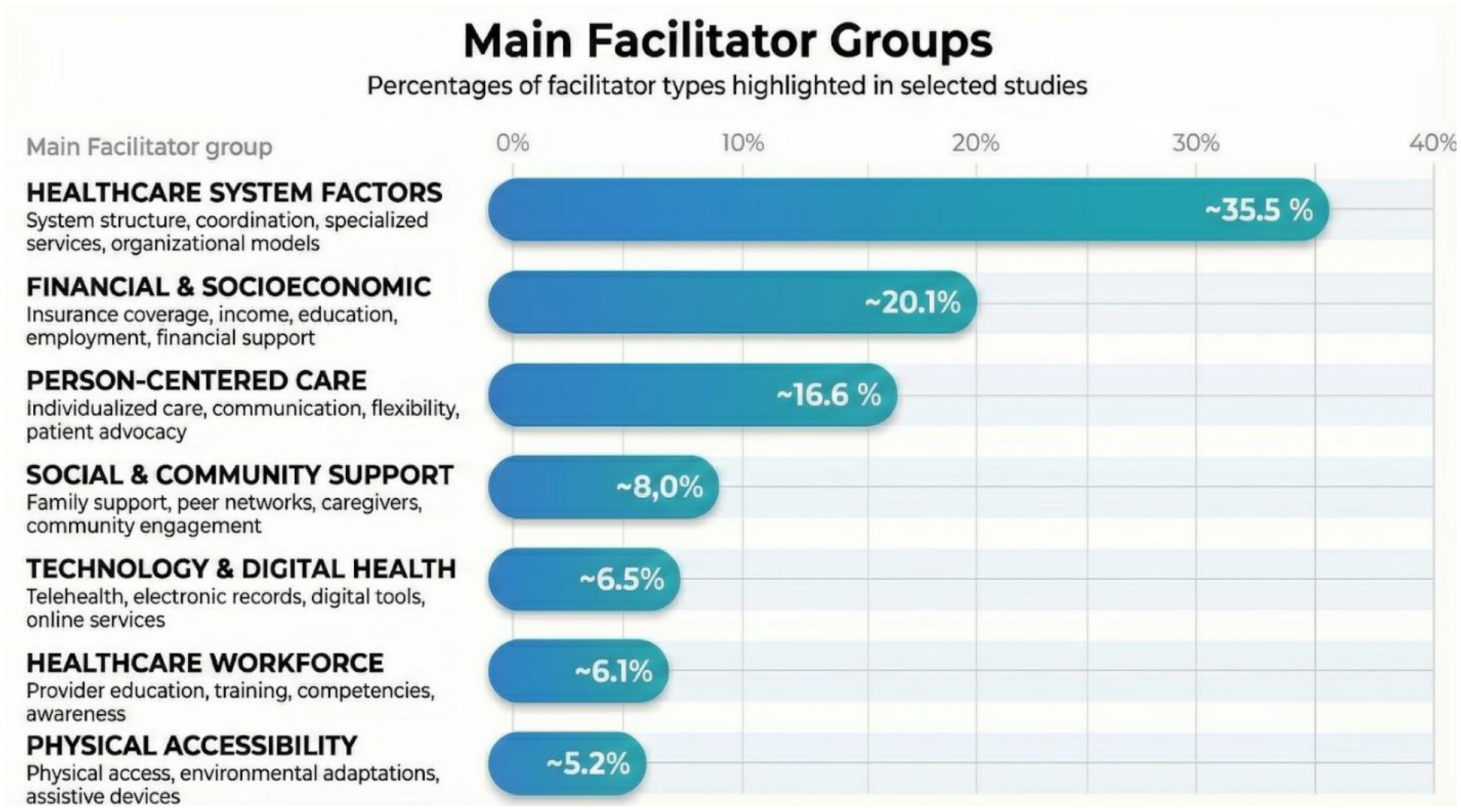
Percentages of access facilitators in selected articles.

We extracted all factors that authors identified as associated with improved healthcare access, which we collectively term “facilitators”. Our approach to data extraction was inclusive and this broad extraction strategy allowed us to capture the full spectrum of evidence, from observed correlations or existing structural features correlated with better access (e.g., insurance coverage, higher income, care coordination structures, provider awareness etc.) to evaluated interventions, purposefully designed programs tested with measured outcomes, including health screening programs, telehealth services, home modification programs, training initiatives. Figure 4 illustrates the distribution of facilitator types identified across the reviewed studies.

When synthesizing findings across all reviewed studies, the most prevalent facilitator was healthcare system factors (14, 15, 17, 63, 64, 83, 84, 87), followed by financial & socioeconomic factors (21, 36, 58, 68, 71, 75, 76) and care coordination/integrated services (39, 42, 52, 59, 67, 76, 87, 98), with smaller contributions from person centered care and social support (8.04%), technology (6.47%), workforce training (6.12%), and physical accessibility (5.24%).

In the context of mental, cognitive, and developmental disabilities, the most prevalent facilitators are as follows: modifications to how healthcare services are structured and provided were reported in 82% of the articles (13, 16–18, 38–46, 51, 53, 54, 57, 67, 73, 86, 87, 97, 98). In 54% of the studies, it is mentioned collaboration between different services and providers (14, 16, 39, 41–45, 53, 54, 57, 67, 73, 87, 98) while in 50% adapted materials and communication methods (13, 16, 38, 40–46, 51, 54, 67, 86) are a source of facilitators.

A review of the literature reveals that financial and insurance support for multiple disabilities is a recurring theme in 76% of articles (19, 20, 29, 31, 34, 36, 64, 68, 75, 79, 81–85, 88, 92, 95), with service delivery models in 60% of articles (29, 31, 47, 60, 65, 75, 88, 92, 95) and person-centered care and support (52%) also being prominent topics (19, 20, 29, 36, 47, 52, 64, 74, 75, 79, 82, 88, 95).

The majority of the physical disability studies reported on identified socioeconomic facilitators (22, 58, 76, 78), followed by the role of social and family support (21, 30, 55, 89–91, 93), and healthcare system infrastructure(25, 37, 56, 59, 77).

### Gaps in literature

The reviewed studies reveal several gaps in the evidence base. In the studies reviewed, researchers inferred facilitators from context as they were not explicitly delineated by the original study authors (13, 14, 17, 18, 36, 43, 45–47, 52, 54, 57, 63, 74, 75, 87, 88), and in fives articles there were no facilitators reported (28, 61, 69, 72, 80). The majority of studies document barriers while most identified facilitators represent observed associations or existing structural features rather than tested interventions. Even though we extracted all factors that authors identified as associated with improved healthcare access under a collective term, analyzed data revealed that 10% of reviewed studies represented evaluated interventions (13, 16, 18, 40–42, 57, 59, 60, 67). The lived experiences of people with disabilities were missing in 10 studies (22, 25, 29, 31, 35, 39, 42, 67, 81, 84) and reported from context in 16 studies (13, 14, 19, 20, 36, 43–47, 54, 57, 63, 75, 87, 88). Caregiver perspectives, on the other hand, were largely absent from the reviewed literature, 58 out of 86 studies did not report caregivers' perspective (15–18, 20–25, 27–36, 38–40, 42, 43, 49, 51, 53, 55, 56, 58, 60–62, 64–72, 74, 76–86, 93, 95, 96). Geographic representation was concentrated in high-income countries, with limited representation from low- and middle-income countries.

## Discussion

The results of this systematic review highlight a complex and multifaceted landscape of health inequities faced by people with disabilities, varying not only by type of impairment but also by context, health system structure, and cultural environment. Systemic and structural barriers were identified in approximately 40% of the reviewed studies, representing a substantial focus within the analyzed literature. The reviewed studies documented healthcare systems characterized by fragmentation requiring extensive coordination, while professional training gaps emerge as critical obstacles identifying poor disability awareness and staff discomfort (41, 42, 52, 54, 67). These findings align with international evidence documenting similar barriers in healthcare access for people with disabilities across diverse country contexts (99–101). Geographic disparities, such as the 64–84-min travel time reported in studied literature for rural populations (75), may reinforce how access appears to be influenced more by geographic location than by medical need alone. However, the geographic concentration of studies in high-income countries creates a generalizability crisis and equity paradox. High-income countries typically have better baseline access, meaning evidence comes from contexts with fewer barriers, while low- and middle-income countries, where disability-related healthcare access barriers are typically more severe and resources more limited remain under-researched (68–70).These findings also align with international studies (7, 8).

Closely related are the disparities in healthcare utilization and access (~27%), identified in our review, which reveal a paradoxical pattern of both overuse and underuse of services. Emergency department overuse is consistent (55, 63) while critical underutilization persists with less than 33% of Parkinson's patients receiving appropriate rehabilitation referrals and 87% of hearing problems remaining untreated (25, 102). The global variation in unmet healthcare needs, ranging from 7% in Switzerland to 62% in Morocco, documented in our reviewed studies, suggests disparities may reflect system-level factors rather than disability-inherent characteristics (70). Analyzing physical disabilities, it is seen that despite decades of disability rights legislation, physical infrastructure continues to present significant barriers. Studies included in our review show this harms body and mind, and reveals how inaccessible environments create physical and emotional distress and disempower negotiations (48, 49, 76).

Communication barriers represent fundamental obstacles consistently documented across diverse disability populations and healthcare settings in our systematic review but also in other studies (13, 21, 25–28, 41, 42, 46, 51, 55, 67, 71, 73, 86, 94, 97, 103–106). These barriers range from basic communication difficulties associated with significant odds ratios, to more complex challenges in symptom reporting among individuals with autism, intellectual disabilities, and sensory disabilities. Technology-mediated communication shows promise, yet the majority of patients remain unaware of available services or unable to access them. These barriers affect not only care delivery but also navigation abilities and meaningful treatment participation. These findings from our systematic review are consistent with broader evidence on poor healthcare service ratings and provider communication challenges experienced by disabled patients (107, 108).

Our review identified that financial barriers creating a vicious cycle with higher cost while having fewer resources leading to medical bill problems and rationing behaviors (15, 24, 66, 69, 75). On the other hand, our reviewed evidence indicates that insurance disparities are systematic, with commercial coverage providing superior outcomes while dual-eligibles experience worst outcomes (35). These barriers create cascading effects where delayed care leads to more expensive emergency interventions, creating downward spirals of health and economic vulnerability. This is further supported by studies included, demonstrating that financial barriers are associated with increased social costs. These costs are mainly attributable to individuals who require medical care and to the decreased productivity of those affected and their families (86).

Health Outcomes and Mortality Disparities also referred as the Ultimate Consequence represent the life-threatening consequence of access barriers documented in our included studies. The reviewed studies documented elevated mortality risks across multiple conditions: higher COVID-19 mortality, cancer disparities including four times higher testicular cancer mortality, and maternal mortality that is 2.3 times higher in women with intellectual and developmental disabilities (17, 44, 45). These findings suggest avoidable suffering, premature death, and preventable secondary disabilities may result from cumulative barriers to care. Differences in treatment and care quality further underscore inequity. This pattern observed in our review is further supported by other studies that document reduced treatment intensity, diagnostic delays, poor care coordination creating fragmented experiences, and deviations from standard protocols, often without clear justification (44, 84, 98, 109, 110), indicating not just access problems but quality concerns when care is accessed. Similarly, technology and digital access present a dual reality: on one hand, digital exclusion further marginalizes those without access to devices or internet; on the other, telehealth, tele rehabilitation, and electronic records have demonstrated strong potential to improve outcomes, reduce emergency visits, and maintain high engagement when inclusive design principles are followed. The reviewed evidence aligns with international evidence suggesting that technology can either widen or narrow disparities depending on its implementation (111–113).

Another significant obstacle that hinders the process of seeking treatment is stigma, described in included studies but also other literature as the fear of being judged by others, but also cultural attitudes, family barriers, and social determinants (40, 53, 72, 73, 114, 115). All these aspects lead to the prevention of certain behaviors (72). Other studies have also shown that these perceptions lead young people to avoid seeking mental health services, thereby delaying intervention and worsening clinical symptoms (116). Similarly, provider and professional factors highlight persistent training gaps, negative attitudes, and resource limitations. The shortage of disability-trained professionals demonstrates how inadequate workforce capacity directly constrains access.

Our research has highlighted a worrying reality in which many identified “facilitators” represent basic accessibility features that should be standard practice such as longer appointments, clear communication, provider awareness (41, 42, 44, 46, 67, 86) rather than enhanced supports. This pattern observed across our reviewed studies suggests that standard healthcare practices may not adequately account for diverse patient needs. The fact that in a number of our studies facilitators were derived from context (13, 14, 17, 18, 36, 43, 45–47, 52, 54, 57, 63, 74, 75, 87, 88) or are missing (28, 61, 69, 72, 80) may reveal a conceptual weakness in how the research approaches enablers of access and studies aren't designed to identify what helps, only what hinders.

Analysis of the included literature revealed that 58 out of 86 studies excluded caregiver perspectives entirely, despite caregivers' critical roles in healthcare access for people with disabilities. Among the 28 studies that included caregiver data, most focused on burden and challenges rather than their insights about effective access strategies or solutions (47, 52, 75, 88, 90, 91, 98). This exclusion in our interpretation represents a significant methodological blind spot. Caregivers often control appointment scheduling, provide transportation, navigate complex systems, and advocate for appropriate care, making their perspectives essential for understanding the full landscape of access barriers and potential facilitators. The absence of their voices means we may be missing major barriers (from a navigator's perspective) and practical solutions that emerge from their lived experience of system navigation.

The reviewed evidence indicates that financial coverage and socio-economic privileges frequently determine access, suggesting the healthcare system may prioritize those with existing advantages rather than compensating for barriers (36, 58, 71). Several studies included, documented that accessing appropriate care often required multiple favorable circumstances converging adequate income, knowledgeable providers, family support, and appropriate technology (13, 52, 67), simply to access what ought to be universal rights. Studies found that healthcare system expansion does not automatically benefit people with disabilities, especially those with multiple disabilities who require integrated services, and may worsen disparities without explicit disability-focused interventions (52, 64, 75, 82). We interpret these findings as indicating that many so-called “facilitators” represent workarounds for systemic gaps rather than genuine enhancements to accessible care. While universal design principles offer critical system-wide improvements, the heterogeneity of disability experiences means that individualized, person-centered accommodations remain essential even within structurally inclusive frameworks, universal design provides the foundation, but cannot eliminate the need for tailored supports.

Finally, and most concerning, intervention and solution effectiveness was the least studied domain. Our analysis reveals an important distinction: most identified “facilitators” represent observed associations or existing structural features rather than tested interventions. Yet these few studies demonstrated encouraging results: home modifications reduced hospitalizations, screening programs exceeded national benchmarks, device loan programs facilitated participation, and community-based services provided viable alternatives to inaccessible hospitals (13, 16, 18, 40–42, 57, 59, 60, 67). These findings underscore the urgent need for a research and policy shift from describing barriers to scaling solutions, improving disability awareness and professional training, facilitating earlier diagnosis, and reducing disparities in treatment. The current evidence base as we interpret it can identify what might help based on associations, but cannot confirm what works through experimental testing. This severe imbalance suggests the field is stuck in a 'diagnostic' phase, we know what's wrong and what's associated with better outcomes, but have limited experimental evidence on what interventions effectively improve access.

## Limitations

In addition to the literature gaps already discussed, our study has specific methodological limitations. Our search was limited to English and Italian-language publication, which excludes potentially relevant research published in other languages. The 2014 publication cutoff, while ensuring contemporary relevance, may have excluded earlier foundational research. We restricted inclusion to adults aged 18 years and older, excluding pediatric populations and the critical transition from pediatric to adult healthcare systems. We did not systematically distinguish between intervention and observed data research during screening, limiting subgroup analyses by evidence type. Sample size variations across studies create challenges in generalizing findings, with underrepresentation of specific groups, cultural variability, and methodological heterogeneity further limiting comparability. Additionally, we synthesized findings across diverse disability policy environments and health system contexts, including countries with different disability rights legislation, healthcare financing models (universal, mixed, private), and levels of accessibility enforcement without systematically accounting for how these policy and system-level differences may shape the barriers and facilitators identified. This variability limits our ability to provide context-specific recommendations.

Despite these limitations, we believe that this review has produced solid evidence that should be taken into consideration for future research and policy development.

Future research should focus on longitudinal, intersectional, and participatory studies to evaluate barriers, interventions, and effective healthcare strategies. Systems, policymakers, and providers must prioritize accessibility, staff training, inclusive policies, and patient-centered care. Without this deeper transformation, the barriers documented in this review are likely to persist, while disabled people continue to suffer preventable health consequences and premature deaths. We acknowledge that future reviews could benefit from a priori classification of evidence types intervention vs. observed facilitators during screening to enable subgroup analyses by methodological rigor. Based on the patterns identified in this review, we argue that the path forward requires healthcare systems to critically examine how current practices may create barriers for people with disabilities.

## Conclusions

This comprehensive review reveals persistent, multifaceted healthcare access barriers for people with disabilities across diverse global contexts at multiple healthcare system levels, with diverse methods and recent focus ensuring timely, relevant findings. Disparities persist across both high- and low-income countries, demonstrating economic wealth does not guarantee health equity. The reviewed evidence shows barriers span six interconnected areas and points to key concerns, despite disability rights legislation, accessibility is typically addressed through individualized accommodations rather than systematic universal design. The medical model overshadows disabling barriers; and patterns of reduced treatment access and delayed care raise questions about priority-setting in healthcare systems. The reviewed studies document that healthcare professionals often lack disability knowledge and hold negative attitudes compromising care quality, while technological solutions retrofit general approaches rather than integrating accessibility, exposing fundamental innovation flaws. These findings highlight that barriers are neither inevitable nor acceptable and cannot be resolved at individual provider or patient levels. Their persistence reflects normalized systemic discrimination, underscoring the need for systemic redesign, political prioritization, and fundamental reframing of disability as central to health equity rather than marginal. Future research should evaluate interventions and effective strategies rather than documenting the same barriers.

## Data Availability

The original contributions presented in the study are included in the article/Supplementary material, further inquiries can be directed to the corresponding author/s.
