# Supplementary material for: Barriers to accessing healthcare for people with disabilities:a systematic review
Source: Front Public Health. 2026 Feb 19;14:1765145. doi: 10.3389/fpubh.2026.1765145 (PMC12960585; doi:10.3389/fpubh.2026.1765145)
Supplement: Supplementary file 1 [file Data_Sheet_1.docx]

**SUPPLEMENTARY SECTION**

**Table S1:** *Data Extraction ( Study characteristics)*

| **Author** | **Year of Publication** | **Nation** | **Study design& Method** | **Study objectives** | **Type of disability** | **Population age** | **Population gender** | **Sample size** | **Type of care service** | **Type of healthcare service** |
| --- | --- | --- | --- | --- | --- | --- | --- | --- | --- | --- |
| Stian H Thoresen(97) | 2017 | Lao People's Democratic Republic (Lao PDR) | Mixed-methods (Survey+Focus groups , Case study) | 1) explore prevalence, 2) assess availability and access | psychic | Participants included both children (6-17 years) and adults (18-59 years), with older adults (60+ years) also surveyed but excluded from intellectual disability analysis | not specified | 2,469 individuals from 939 households for the screening survey; 6 representatives from key government agencies, disabled people's organizations (DPOs), and service providers for the focus group | Education, health, social, and rehabilitation services | Private |
| Avra Selick(67) | 2018 | canada | Qualitative study(Interviews) | 1) barriers 2) facilitatotors | psychic | Adults | Not specified | Not specified | Primary healthcare | Universal |
| Charlotte Featherstone, (38) | 2022 | UK | Quantitative, cross-sectional survey | Investigate healthcare barriers, well-being, and intersectional inequalities | psychic | Adults (18+) | 50.8% female, 35.2% male, 13.3% non-binary/other | 128 initial, 42 follow-up | Primary healthcare | Universal |
| Arnold (51) | 2024 | Australia | Quantitative, cross-sectional survey | 1)Explore barriers to access, compared with non-autistic 2)Identify factors associated with barriers | psychic | Adults (25+ years) | 57.41% female, 34.60% male, 7.98% gender-diverse (in autistic group); 77,14% female, 22,86% male in non autistic) | 333 total (263 autistic, 70 non-autistic) | Primary healthcare | Mixed |
| Sebastian Dern,(86) | 2016 | Germania | Qualitative study( Meetings+ Discussion) | 1) barriers to healthcare 2) strategies to overcome | psychic | Adulti (>18 anni) | Male+female | Not specified (23 regular meetings of autistic adults and professionals) | General health services, hospital care, emergency services, and primary medical care | Universal |
| Nicole David, (71) | 2024 | Germania | Quantitative,cross-sectional survey | 1) investigate quality of life 2)analyze predictors including healthcare barriers | psychic | Adults (mean age 38.9 years) | 55.3% female, 36.3% male, 8.4% gender-diverse | 311 | General healthcare services | Universal |
| Ahmed A. Mohamed, (72) | 2024 | Saudi Arabia | Quantitative cross-sectional study(Interviews) | 1) evaluate personal, family, societal, and medical barriers to consultation and treatment, 2)gender variation in barriers | Psychic | 18 years and older | Both males (40.2%) and females (59.8%) | 463 individuals | Mental health consultation and treatment services | Universal |
| Fintan Sheerin,(98) | 2024 | Ireland | Mixed methods( Surveys+Qualitative methods). | 1) examine the care and service options | psychic | Older adults (study defined as 40 years and older) | Both males and females | 77 participants (32 senior managers, 17 direct care staff, 9 older people with intellectual disabilities, 5 family members of current service users, 3 family members of deceased service users) | Community-based residential services, intellectual disability services, nursing homes, day services, and retirement options | Universal |
| Laura Hughes-McCormack,(39) | 2020 | United Kingdom (Scotland) | Quantitative ,cohort study (Data records) | 1)quantify trends in good practice 2)healthcare inequalities gap | psychic | Adults (18+ years) | Both males and females (55.2% males in 2007-2010 and 58.0% males in 2014) | 721 adults with intellectual disabilities in 2007-2010 and 3,638 in 2014, compared with general population data for 764,762 adults in 2007 and 799,893 in 2014 | Primary healthcare | Universal |
| R.S. Balogh,(63) | 2015 | Canada (Ontario) | Quantitative,cohort study(Data analysis) | 1)Compare diabetes prevalence, incidence, hospitalizations | psychic | Adults aged 30-69 years | both female and male | 28,567 adults with IDD and 2,261,919 adults without IDD | Primary care | Universal |
| Maji Hailemariam,(87) | 2016 | Ethiopia | Qualitative study(Interviews+focus groups) | 1)barriers 2)strategies to overcome | psychic | Adults | Both male and female (though most participants were male, with only 5 out of 22 in-depth interviews being with women) | 33 (21 in-depth interviews, 12 focus group participants) | Primary mental healthcare | Universal |
| Melissa L. Desroches,(13) | 2024 | US | Qualitative study(Surveys+Interviews) | 1) access barriers 2)health equity 3)practicies and guidelines | psychic (Developmental disabilities (cognitive)) | Adults (18 years and older) | Not specified | 44 expert panelists | Telehealth care | Mixed |
| Janet Golder,(57) | 2024 | Australia | Quantitativecohort study(Record review) | 1) design care model, 2) create interventions, 3) assess sedation, 4) analyze participants | psychic- Intellectual and developmental disabilities (cognitive) | Adults Adults (18+ years), median age 26 (median age 26 years, range 18-71 years) | Both male (75.6%) and female (24.4%) | 127 | Procedural care, sedation services | Universal |
| Errol Cocks,(52) | 2014 | Australia | Qualitative study(Interviews) | 1) document experiences, 2) identify recovery factors, 3) assess impacts on daily life, 4) determine needs, 5) guide policy, | mental and physical (acquired brain injury and mental illness, primarily depression) | Adults (late teens to early fifties) | Both (7 males, 1 female) | 15 participants (8 people with ABI/MI, 2 family members, and 5 support workers) | Rehabilitation, mental health, and disability support services | Universal |
| Lisa A. Razzano,(14) | 2014 | US | Quantitaitve,cross-sectional survey (Interviews) | 1)Examine prevalence of medical conditions and factors associated 2)Assess treatment rates 3)Compare to general population 4)Explore demographic correlates 5)Investigate healthcare disparities | psychic | Adults (mean age 46.5 years) | Both (48.7% female, 51.3% male) | 457 adults with serious mental disorders | Medical and mental healthcare services, particularly regarding treatment prevalence for co-occurring physical health conditions | Mixed |
| Amy Oliver,(40) | 2024 | UK | Qualitative study(Interviews+Questionnaires) | 1) examine impact on mental health access, 2) understand barriers/facilitators to accessing digital technology, 3) explore impact on wellbeing, 4) identify implementation lessons. | psychic | Adults (21-88 years, mean 52.42) | both gender (in the interview sample of 12 participants: 50% female, 42% male, 8% gender fluid/queer) | 12 participants for interviews (from 83 service users who engaged with the scheme) | Mental health services with remote care delivery, including telehealth practices and video consultations | Universal |
| Kevin Lu, (15) | 2022 | US | Quantitattive ,cross-sectional survey | 1) track financial barriers, 2) health outcomes, 3) measure utilization effects, 4) determine costs. | psychic (Cognitive impairment) | Adults aged 18 years or older | Both genders (54.9% male, 45.1% female without barriers; 57.3% male, 42.7% female with barriers) | 35,773,286 weighted respondents with cognitive impairment (nationally representative) | Primary care | Mixed |
| Sarah Wigham,(41) | 2022 | UK | Qualitative study(Focua grups+Interviews+Survey) | 1) investigate stakeholder perspectives primary care access, 2) address disparities, 3) formulate recommendations. | psychic | Adults (18+ years) | both female and male | 16 participants (4 people with learning disabilities, 4 relatives, 8 professionals) | Primary care | Universal |
| Michael Brown,(42) | based on reerences 2016-2017 | UK | Qualitative study(Interviews) | 1) examine practitioner perspectives on diabetes/ID, 2) identify care barriers, 3) create service improvements. | psychic (Intellectual disability) | Adults | no | 29 participants (14 ID practitioners, 10 diabetes practitioners, 5 community care staff) | Diabetes care in primary and secondary care | Universal |
| Rafat Hussain, (53) | 2020 | Australia(with collaboration from USA) | Quantitative,cross-sectional survey(Interviews) | 1) examine burden 2) identify sociodemographi  c correlates, 3) assess health conditions by age/sex. | psychic ( Intellectual disability) | Adults 60+ years (range 60-87 years) | Both males (62.7%) and females (37.3%) | 391 individuals (236 urban/155 rural) | Community-based health services | Universal |
| Laura McKernan Ward,(43) | 2024 | Scotland (UK) | Quantitative cohort study(Record linkage) | 1) examine cancer mortality rates compared to incidence, 2) identify cancer pattern differences between ID and general population, 3) determine specific cancer risks. | psychic | Adults aged 18+ years (mean age 43.9 years for intellectual disability cohort, 49.0 years for general population) | Both males (55.6% of ID cohort) and females (44.4% of ID cohort) | 17,203 adults with ID linked to 566,061 general population controls | Cancer detection and treatment services | Universal |
| J.D. Reinhardt (74) | 2020 | China | Quantitative,cross-sectional study(Interviews) | 1) measure disabled survivors' PTSD rates, 2) evaluate limitations/barriers, 3) analyze relationships. | Physical and neurological impairments | Adults (16+ years, mean age 61.8) | Both males (34.26%) and females (65.74%) | 289 | Community-based rehabilitation, mental health services | Mixed |
| Dan Qiu,(73) | 2024 | China | Quantitiative ,cross-sectional study(Interviews) | 1) examine mental health service policies, 2) assess service coverage, 3) evaluate user outcomes. | psychic | Mean age of 47.1 years (SD: 13.3) | 45.9% male, 54.1% female | 972 patients | Primary care | Universal |
| E. Sally Rogers,(16) | 2016 | US | Quantitative,cohort study (Interviews) | 1) integrate primary care, 2) improve healthcare access/coordination, 3) evaluate nurse practitioner intervention | psychic | Mean age of 43.5 years (SD=10.9) | 67% female, 33% male | 200 individuals (94 assigned to experimental group, 106 to control group) | Integrated primary care (nurse practitioner in mental health setting) | Mixed |
| Monika Mitra (17), | 2021 | US | Quantitative,cross-sectional study (Sample Data) | 1) compare maternal outcomes (morbidity and mortality), 2) find risk factors, 3) analyze disability impacts. | psychic | Mean age 27.5 years (primarily reproductive age women; age distribution by category: <20 years (12.7%), 20-24 years (28.9%), 35-39 years (50.4%), ≥40 years (8.1%)) | 100% female | 32,324 deliveries to women with intellectual and developmental disabilities and 96,972 deliveries to women without intellectual and developmental disabilities (matched comparison group) | Hospital-based delivery care and maternal health services | Mixed |
| Marisa Brown, (18) | 2016 | US | Quantitative,cross-sectional study(Record review) | 1) assess adherence to ID screening adherence, 2) identify barriers, 3) develop preventive health policies | psychic (intellectual disability) | Range 42-90 years, median age 59 years | 63.5% male, 36.5% female | 444 individuals with intellectual disability (out of 501 eligible Evans class members, with a response rate of 88.6%) | Preventive health screening services | Universal |
| Charlotte Emily Mott,(44) | 2019 | UK | Qualitative case report(Literature review) | 1) identify disabled cancer care challenges, 2) show adaptations, 3) promote patient involvement, 4) examine MDT legal frameworks. | psychic (Learning disability (LD)) | 61-year-old | Male | 1 (single case report) | Oncology, chemotherapy, surgical treatment | Universal |
| Natasha Layton,(54) | 2024 | Australia | Qualitative study(Workshops) | 1) explore dementia rehabilitation barriers, 2) identify access solutions. | psychic (dementia) | Adults | no | 26 participants (5 people living with dementia, 8 care partners, and 13 health professionals) | Rehabilitation services | Universal |
| R Asaad Baksh, (45) | 2021 | UK | Quantitative,cohort study(Paper case report form) | 1) compare COVID hospitalization experiences, 2) detect disparities, 3) assess resource access Compare symptoms, complications and mortality | psychic (Intellectual disability) | All age groups (predominately adults over 40 years, with only 25% under 40) | Both (56.5% male, 43.5% female) | 506 patients with intellectual disabilities matched with 1,518 controls (1:3 ratio) | Hospital care, intensive care, respiratory support interventions | Universal |
| David Mason, (46) | 2022 | uk | Mixed,cross-sectional study( Questionnaire)+ | 1) gather health check perspectives, 2) identify key design elements, 3) determine access factors. | psychic(Autism) | Adults (76.4% between 26-60 years) | Both males (40%) and females (53.3%), with 3% not reporting as male or female | 458 participants (441 autistic adults and 17 proxy responders) | Primary care health checks | Universal |
| Jill Bradshaw,(47) | 2024 | UK | Mixed-methods (Survey) | 1) explore COVID impact 2) examine access issues, 3) identify health effects, 4) determine helpful supports | psychic and physical (Profound intellectual and multiple disabilities (PIMD)) | 16-69 years (Mean: 30.6, SD: 11.9) | Both males (81) and females (80) | 166 participants (145 family carers, 21 paid carers) | Day services, respite care, community activities, health services | Universal |
| Dr Jean-Francois Trani (75), | 2017 | Afghanistan | Quantitative,cross-sectional survey | 1) evaluate investment effects on access, 2) identify perception factors, 3) assess needs coverage | multiple disability (physical, sensory, mental and associated) | Media di 31,1 anni (DS 21,0) nel 2005 e 30,9 anni (DS 21,3) nel 2013 | 61% male, 39% female | 1,245 people with disabilities (334 in 2005 survey, 961 in 2013 survey) | Basic Package of Health Services (BPHS) | Miixed system |
| Veronika Reichenberger,(88) | 2024 | brasile | Qualitative study (Interviews) | 1) gather disabled access views, 2) identify factors, 3) compare regions | General disability Various (physical, visual, hearing, intellectual impairments) | Adults (18 years and older), with 9 participants aged ≥60 years and 35 participants aged <60 years | Both males (19) and females (25) | 44 participants | Primary healthcare services | Universal |
| Carrie E. Henning-Smith, (19) | 2016 | US | Quantative .cross-sectional survey | 1) Compare medical care access by disability status and household composition 2) Assess if these factors interact | multiple disability | Adults aged 65 and older | Both males and females (56.2% female in the full sample) | 16,720 participants | Primary medical care | Mixed |
| Stephanie E Rogers, (36) | 2015 | US | Quantitative, cohort study(Survey data) | 1) analyze discrimination-disability links, 2) measure elderly discrimination rates. | multiple disability | Adults over 50 years (mean age 67 years) | Both (56.3% female, 43.7% male) | 6,017 adults | General healthcare services | Mixed |
| Nancy A. Miller, (20) | 2014 | US | Quantitative, cross sectional study(Survey Data ) | 1) analyze demographic healthcare gaps, 2) compare age-related differences. | Multiple disability types | Middle-aged (31-64) and older (65+) adults | Both males (44.54%) and females (55.46%) | 13,174 adults with disabilities (7,768 middle-aged and 5,406 older adults) | Primary care, preventive services | Mixed |
| Mohammad Hifz Ur Rahman,(68) | 2019 | Multiple countries - China, Ghana, India, Mexico, Russia, and South Africa | Quantitative ,cross-sectional survey | 1) assess outpatient disparities, 2) compare countries, 3) analyze elderly patterns, 4) identify determinants | Multiple types | Older adults (50+ years) | Both males and females (varied by country) | Total of 34,098 older adults across all countries (China: 13,158; Ghana: 4,305; India: 6,560; Mexico: 2,301; Russia: 3,938; South Africa: 3,836) | Outpatient healthcare services | Mixed |
| Hussain Zandam,(76) | 2019 | Nigeria | Quantitative,cross-sectional study(Questionnaire) | 1) Compare healthcare accessibility perceptions between people with/without physical disabilities, 2) analyze access barriers, 3) examine contextual factors, 4) identify equity issues | Physical disabilities | Adults (primarily 16-49 years, with 9% over 50 years) | Both males (52%) and females (48%) | 426 total (213 with physical disabilities, 213 without disabilities) | General healthcare services (primary health centers, hospitals, private clinics) | Mixed |
| Sara H Rotenberg,(77) | 2024 | Sub saharan Africa | Quantitative ,cross-sectional(Survey Data) | 1)barriers | physical | 18-49 | female | 10,021 ( 306 with disabilities) | Maternal ,prenatal,postnatal and postpartum care | Universal |
| Ali Lakhani,(56) | 2022 | Australia | Quantitative,,cohort(Survey) | 1)access of health services,2) health conditions, 3)quality of life, 4) autonomy | physical(spinal cord injury) | Mean age 55.81 | Mixed | 71 participants(52 males, 18 females) | Primary care, specialist care, rehabilitation, allied health services | Universal |
| Ana Oña,(69) | 2023 | International(22 countries from America,Africa,Asia,Australia,Europe) | Quantitative,cohort study(Survey) | 1) health differences 2) impact factors on health inequalities | physical(spinal cord injury) | mean ages 51 years | mixed | 11529 in total | General healthcare services | Universal |
| Claire Ryan,(21) | 2018 | United States | Quantitative,cross-sectional(Survey) | 1)eHealth-provider communication association | Physical(hearing loss) | mean age 42 years | mixed | 515 | Primary healthcare communication | Universal |
| Bougangue Bassoumah,(89) | 2019 | Ghana | Qualitative study(Interviews) | 1)the socio-cultural challenges | physical | 22-45 years old | Female | 40 (20 with disabilities and 20 without disabilities) | Maternal and neonatal healthcare services | Universal |
| Jihyun Kwon,(83) | 2019 | Korea | Quantitative,cohort study(Database review) | 1) diagnosis, 2)treatment patterns, 3) survival outcomes | multiple | adults | mixed | 4,090 (809 with disabilities, 3,281 without disabilities) | Specialist cancer care, dialysis treatment, autologous stem cell transplantation | Universal |
| Reuben Kalavina,(90) | 2019 | Malawi | Qualitativestudy (Interviews+Focus groups) | 1)challenges during and after rehabilitation2)experiences | Physical | Not specified | Mixed | 18 participants (9 stroke patients and 9 spouses) | Rehabilitation services | Mixed |
| Fareha Nishat,(64) | 2021 | Canada | Quantitative,cohort study (Administrative Data) | 1) prenatal care adequacy | Multiple | 15-49 years | Female | 1,069,388 (83,752 physical disabilities, 25,685 sensory disabilities, 1,219 intellectual/developmental disabilities, 4,966 multiple disabilities, 953,766 without disabilities) | Prenatal care | Universal |
| Carmen E Capo-Lugo,(22) | 2019 | United States | Quantitative,cohort study(Record review) | 1)factors affecting receipt of therapy consultations/interventions | Physical disability (stroke-related) | Mean age 62.1 years | mixed | 1366 participants(54.2% women, 45.8% men) | Acute care hospitalization | Mixed |
| Kavita Makan,(58) | 2024 | South Africa | Quantitaitve,cross-sectional (Survey) | 1) unmet needs 2)patient journey 3)functional disability, | Physical(musculoskeletal - axial spondyloarthritis) | Mean age 44.7 years | Mixed | 146 total | Rheumatology specialty care | Mixed |
| Robert Kokou Dowou, (91) | 2023 | Ghana | Qualitative study(Interviews) | 1)coping strategies 2) roles of caregivers | Physical | <30 to 60+ years old | mixed | 33(25 patients and 8 caregivers) | Outpatient chronic disease management | Universal |
| Barbara Kołłątaj,(78) | 2015 | Poland | Quantitative,cross-sectional (Survey) | 1)rehabilitation equipment needs | physical(primarily mobility impairments) | Not specifically defined | Mixed | 478 disabled individuals | Specialist care | Universal |
| Rajeswaran Thiagesan,(92) | 2024 | India | Qualitative study(Interviews) | 1)diabetes self-management challenges 2)influencing factors | multiple | middle-aged and elderly | mixed | 16 people(9 women and 7 men) | Diabetes self-management in community and primary healthcare | Mixed |
| Elise‐Marie Dilger,(93) | 2024 | Germany | Qualitative study(Interviews) | 1)Mobility devices | Physical (multiple sclerosis with gait impairment) | 33-76 years old | mixed | 19 with disabilities ( 12 females, 7 males ) | Rehabilitative services, mobility assistive devices provision | Universal |
| Olena Bychkovska,(70) | 2023 | international study (Africa,Asia,Australia, Europe ,South and North America ) | Quantitativecross-sectional (Questionnaire) | 1)association between health system characteristics and access | Physical(spinal cord injury) | >18 years old | mixed | 12,588 participants | General healthcare services, multidisciplinary services | Mixed |
| Rosemary B Hughes,(23) | 2022 | United States | Qualitative study(Focus groups) | 1)health information seeking pathways 2) barriers | Physical | 22-72 years old | women | 21 | Health information access and resources | Not specified |
| Michelle M. Y. Wong,(65) | 2022 | Canada | Qualitative study(Interviews) | 1)pandemic impact on wellbeing, health, care delivery 2)virtual services | multiple(Spinal Cord Injuries and Acquired Brain Injuries) | 36-75 years old | Mixed | 11( 7 females, 4 males) | Rehabilitation services, telehealth/virtual healthcare, homecare, outpatient services | Universal |
| Seon Mee Park,(85) | 2022 | South Korea | Quantitative, cohort study(Database analysis) | 1)disability impact | multiple | Primarily adults | Mixed | 15,065(3,693 patients with disabilities and 11,372 patients without disabilities) | Cancer diagnosis, treatment | Universal |
| Tare Lowe,(94) | 2024 | New Zealand | Qualitative study(Intewrviews) | 1)hearing healthcare access 2)hearing assessment | physical(hearing loss) | 30-70 years old | Mixed | 8 (4 male,4female) | Ear and hearing healthcare services | Mixed |
| Niu Tian,(24) | 2022 | United States | Quantitative,ccross-sectional Survey | 1)access to health care | physical(epilepsy) | ≥18 years | Mixed | 60,281 (735 active epilepsy, 456 inactive epilepsy, 59,090 without epilepsy) | Multiple services | Mixed |
| Dikaios Sakellariou,(48) | 2019 | United Kingdom | Qualitative study(Interviews) | 1)barriers | Physical | 35-75 years range | Mixed | 18 (10 female, 8 male participants) | Cancer services | Universal |
| Verusia Chetty,(59) | 2016 | South Africa | Qualitative study(Focus group) | 1)barriers of access | physical | 20-60 +years(1 partecipant over 60) | Mixed | 30(21 females, 9 males) | Rehabilitation services | Universal |
| Amber Willink,(25) | 2021 | United States | Quantitative,cross-sectional Survey | 1)demographics, 2)utilization patterns, 3)alternative pathways | physical(hearing loss) | Not specified | Mixed | 7,361(all medicare beneficiaries) | Hearing care services and alternative delivery models | Mixed |
| Whitney Powell,(26) | 2019 | United States | Qualitative study(Interviews) | 1)impact of hearing loss 2) barriers | Physical(hearing loss) | 50-78years | mixed | 40 | Hearing healthcare services | Mixed |
| Mansha Mirza,(27) | 2020 | United States | Qualitative study(Focus groups) | 1)clinician and patient experiences 2)care impact | Physical | 38-82 years | mixed | 20(16 female and 4 male) | Rehabilitation services | Mixed |
| Ruth Bailey,(49) | 2019 | United Kingdom | Qualitative study (Interviews) | 1)barriers 2)effects | Physical | 18-65 years | Mixed | 27 | Hospital care | Universal |
| Sarah E. Bauer,(28) | 2016 | USA | Quantitative,cross-sectional Survey | 1) physical and communication barriers | multiple | >18 years | Mixed | 1,429 Florida residents (471 with disabilities, 958 without disabilities) | General healthcare services access v | Mixed |
| Kevin J. Bennett,(29) | 2016 | USA | Quantitative,cross-sectional (Data analysis) | 1)diabetes service utilization | multiple | >18 years(primarily 44+ years) | Mixed | 173,678 total respondents | Primary care, preventive services (diabetes management) | Mixed |
| Neelam Borade,(96) | 2019 | India | Qualitative study(Interviews) | 1)experiences 2)barriers/facilitators,3)post-use life changes | Physical | 18-55 years | Mixed | 25(11 males and 14 females) | Rehabilitation services | Mixed |
| Katherine Froehlich-Grobe,(31) | 2016 | United States | Quantitative cross-sectional(Program data)) | 1)utilization 2)compliance 3)follow-up | Multiple | 21-65+ years | Female | 7,521 women total(864 with disabilities) | Preventive cancer screening services | Mixed |
| Jennifer L. Wong,(30) | 2019 | United States | Quantitative ,cross-sectional (Survey) | 1)utilization, accommodations and barriers 2) reasons of delays | Physical | 29.37-100.17 years | Mixed | 1,159 | Various medical providers, rehabilitation services, preventative care | Mixed |
| Daniel R. Terry,(55) | 2015 | Australia | Mixed methods(Questionnaire+Interviewss+Focus groups) | 1)health awareness 2)access 3)ways to enhance interaction | physical(hearing loss) | 22 -82 years | Mixed | 43 | Primary care, emergency services, mental health services | Universal |
| Astha Singhal,(32) | 2016 | United States | Quantitative,cross-sectional study(Survey) | 1)disparities in acces 2) role of psychological distress | Physical(mobility impairmant) | ≥18 years | Mixed | 36,697 (2,170 with mobility impairment 34,527 without mobility impairment) | Dental care and medical care | Mixed |
| Dong Wook Shin,(84) | 2021 | South Korea | Quantitative cohort study(Database linkage) | 1)disparities | Multiple | 66-75 years | Male | 42,112 total (7,924 with disabilities, 34,188 without disabilities) | Cancer diagnosis, treatment and survival outcomes | Universal |
| Luz Mairena Semeah,(33) | 2021 | United States | Quantitative, cross-sectional study (Administrative Data) | 1)health service utilization | Physical | mean age 72.5 years | Mixed | 29,910 | Home modification services, hospitalization, outpatient care | Universal |
| Dikaios Sakellariou,(50) | 2019 | United Kingdom | Qualitative study (Interviews) | 1) experiences 2)discrimination | Physical | adults | Mixed | 18 (10 women, 8 men) | Cancer care services | Universal |
| Janet Pearson,(79) | 2020 | New Zealand | Quantitative ,cross-sectional (Survey) | 1)access | multiple | 20–70 years | Women | 84 | Preventive healthcare services | Universal |
| Jill Hanass-Hancock,(60) | 2014 | South Africa | Mixed methods(Workshops+Surveys+Interviews) | 1)workshop impact | Multiple | Not specified | mixed | 60(15 male and 45 female ) | HIV prevention, care, treatment and support services | Universal |
| Aviwe S Mgibantaka,(62) | 2024 | South Africa | Quantitative cohort study(Medical records) | 1)survival rate | Physical(lower extremity amputations) | Mean age 60.7 years | Mixed | 439 | Tertiary hospital care | Universal |
| Namkee G Choi,(34) | 2020 | United States | Quantitative,cross-sectional (Survey) | 1)insurance coverage impact on healthcare access | multiple | 50–64 years | mixed | 49,025 | Primary , specialty, emergency, hospitalization, dental and eye care | Mixed |
| Elham Mahmoudi,(35) | 2024 | United States | Quantitativecohort study(Data analysis) | 1)comparison of preventive care/preventable hospitalizations | Physical | .> 18 years | Mixed | 56,831 | Preventive care services and preventable hospitalizations | Mixed |
| Andrew Sentoogo Ssemata,(95) | 2024 | Uganda | Qualitative study(Interviews) | 1)interventions healthcare barriers | Multiple | 18-80 years | Mixed | 27 (14 females, 13 males) | Primary healthcare services | Mixed |
| Manjula Marella,(80) | 2016 | Philippines | Quantitative,ross-sectional (Survey) | 1) prevalence 2)access | multiple | >18 years | Mixed | 2,904 | Community services, healthcare, rehabilitation, social welfare, education, employment | Mixed |
| Shikha Gupta,(66) | 2019 | Canada | Qualitative study(Interviews) | 1)strategies 2)factors 3)access | Physical (spinal cord injuries) | median age of 57.5 years | Mixed | 12( 8 females, 4 males) | Outpatient medication management | Universal |
| Francesca M Nicosia,(37) | 2018 | United States | Qualitative study(Interviews) | 1) barriers 2)facilitators | Physical | Older adults | Mixed | 24 primary care provider | Primary care | universal |
| Shengxuan Jin,(82) | 2024 | China | Quantitative,cross- sectional (Database) | 1) health services utilization 2) inequalities | multiple | >45 years old | Mixed | 9,795 with disabilities | outpatient services, inpatient services, and self-treatment services | Mixed system |
| Badriyeh Karami,(81) | 2024 | Iran | Quantitative, cross-sectional (Questionnaire) | 1) determinants 2)inequalities | Multiple | >18 years | Mixed | 766 | Inpatient hospitalization | Mixed |
| Jacqueline Moodley,(61) | 2015 | South Africa | Quantitative, cross-sectional (Survey data) | 1)comparison of health outcomes and access | Physical | >18 years (mean: disabled=47, non-disabled=34) | Mixed | 14,491(2,944 with disabilities, 11,547 non-disabled) | General health care services | Mixed |

**Table S2:** *Data Extraction ( study objectives and outcomes)*

| **Author** | **Principal results/Outcomes** | **Facilitators** | **Barriers** | **Experiences of individuals with disabilities** | **Experiences of caregivers** |
| --- | --- | --- | --- | --- | --- |
| Stian H Thoresen | 1) poor professional disability awareness, 2) no local diagnostics, 3) limited public understanding, 4) insufficient funding, 5) poor service coordination, 6) inadequate policies, 7) few services, 8) foreign diagnostics required, 9) parent-led initiatives dominate. | 1) parent advocacy/leadership, 2) parent associations like AfA, 3) parent-led service development, 4) international training in neighboring countries, 5) school partnerships for inclusion, 6) international funding for parent organizations, 7) growing government recognition of intellectual disabilities. | 1) social/cultural stigmatization, 2) superstitious beliefs about disability causes, 3) limited budget allocation, 4) no in-country diagnostic expertise, 5) absent specialist training, 6) focus on physical over intellectual disabilities, 7) reliance on expensive neighboring-country services, 8) poverty limiting access, 9) legal impediments excluding disabled from birth in social security laws. | 1) poor recognition without visible signs, 2) service exclusion favoring acquired disabilities, 3) minimal education for severe cases, 4) heavy caregiver burden, 5) behavior management difficulties, 6) stigma-driven isolation, 7) limited employment/independence prospects, 8) lifelong parental dependence, 9) restricted community involvement. | 1) Required Thailand travel for diagnosis, 2) minimal-support home management for years, 3) social stigma/blame, 4) leadership in service development, 5) parent association formation, 6) personal resource investment, 7) international staff training procurement, 8) adaptation to age-related challenges, 9) aging-parent concerns about long-term care. |
| Avra Selick | 1) prioritization difficulties for small complex population, 2) inadequate IDD training, 3) poor patient recognition, 4) staff discomfort, 5) competing time demands, 6) inconsistent implementation, 7) routine integration problems, 8) unsustainable changes, 9) limited skill practice opportunities. | 1) strong healthcare champions, 2) organizational culture alignment, 3) electronic prompts/point-of-care tools, 4) research/improvement experience, 5) national guidelines (especially primary care), 6) local data demonstrating need, 7) intervention value evidence sharing, 8) interprofessional implementation teams, 9) adequate implementation resources/time | 1) low IDD prevalence reducing focus, 2) high patient complexity, 3) resource/time constraints, 4) competing priorities, 5) staff turnover/morale issues, 6) passive leadership, 7) poor organizational change timing, 8) missing physician incentives, 9) staff shortages, 10) poor community-hospital relations. | no | No |
| Charlotte Featherstone, | 1) Total barriers remained stable across pandemic, 2) Nature of barriers shifted, 3) Pre-pandemic barriers: waiting rooms, emotional concerns, provider misinterpretation, 4) During/post-lockdown barriers: appointments, follow-up, communication, 5) Remote healthcare increased from 23.4% to 40%+, 6) Barriers persisted after restrictions eased, 7) Autistic adults showed poorer well-being than general population | 1) Remote healthcare improved access for some, 2) Stable routines during lockdown, 3) Reduced social demands, 4) Multiple contact methods, 5) Online booking, 6) Choice in communication methods | 1) Provider communication difficulties, 2) Follow-up problems, 3) Appointment challenges, 4) Telephone use, 5) Sensory issues, 6) Provider knowledge gaps, 7) Behavior misinterpretation, 8) Healthcare system navigation, 9) Executive function challenges, 10) Symptom reporting difficulties | 1) Barriers varied by gender, education, additional disabilities, 2) Females/non-binary more likely misinterpreted, 3) Additional disabilities created more communication problems, 4) Lower education linked to comprehension barriers, 5) High anxiety accessing healthcare, 6) Barriers associated with decreased well-being | No |
| Samuel RC Arnold, | 1) Autistic adults reported significantly more barriers to healthcare (4.58) than non-autistic adults (0.76), 2) 79% of autistic participants reported at least one barrier, 3) 19% reported eight or more barriers, 4) Key barriers included handling waiting rooms, trouble following-up on care, difficulty reporting symptoms, emotional barriers, appointment challenges, and communication problems | Not extensively discussed, but recommendations included in discussion: 1) National roadmap for improving healthcare, 2) Co-designed policies with autistic adults, 3) Environmental adaptations to health facilities, 4) Improved provider education, 5) Enhanced social support networks | 1) Fear and anxiety impacting primary care access, 2) Trouble following up on care, 3) Difficulty understanding healthcare information, 4) Problems making appointments, 5) Communication difficulties, 6) Sensory discomforts, 7) Cost concerns, 8) Misinterpreted behaviors, 9) Not being taken seriously by providers, 10) Inadequate social support | 1) Gender-diverse autistic adults faced additional barriers, 2) Higher anxiety levels associated with increased barriers, 3) Greater disability associated with more access problems, 4) Lower satisfaction with social support linked to more barriers, 5) Significant co-occurring mental health conditions, 6) Difficulties with waiting rooms and sensory environments | No |
| Sebastian Dern, | The study identified numerous barriers to healthcare including: 1) difficulties making appointments by phone, 2) sensory overstimulation in waiting areas, 3) discomfort with unannounced touch during examinations, 4) communication difficulties with providers, 5) stress due to open questions and lack of time to respond, 6) poor understanding of medical staff about autism, 7) difficulty with staff changes, 8) problems expressing pain or other symptoms, 9) difficulties understanding healthcare systems | 1) Making appointments via email or fax, 2) scheduling earliest or latest appointments to reduce waiting, 3) providing separate waiting areas, 4) announcing physical contact before examination, 5) providing written information, 6) using clear, concrete language, 7) avoiding irony or figures of speech, 8) allowing extra time to respond, 9) using visual aids, 10) providing consistent staff members | 1) Sensory issues (lights, sounds, smells), 2) communication problems, 3) discomfort with physical contact, 4) stress due to uncertainty about waiting times, 5) difficulty filling out paperwork, 6) misinterpretation of behavior by medical staff, 7) being overlooked or not taken seriously, 8) difficulty reporting symptoms, 9) face blindness (not recognizing staff), 10) literal understanding of language | 1) Higher rates of mental and physical health conditions compared to general population, 2) greater difficulties accessing healthcare, 3) susceptibility to having unmet healthcare needs, 4) increased risk of using emergency services, 5) challenges with both verbal and nonverbal communication in medical settings, 6) difficulties translating healthcare information into concrete actions, 7) prosopagnosia creating anxiety about not recognizing staff | No |
| Nicole David, | 1) Autistic adults reported significantly reduced mental and physical health-related quality of life compared to general population, 2) Mental health-related quality of life was particularly affected (below average), 3) Barriers to healthcare significantly predicted both mental and physical health-related quality of life, 4) More barriers predicted poorer quality of life, 5) Barriers emerged as the strongest predictor for mental health-related quality of life | 1) Employment status (being employed predicted better mental health-related quality of life), 2) Higher educational attainment (high school diploma or equivalent predicted better physical health-related quality of life), 3) Earlier diagnosis of autism, 4) Lower rates of mental and somatic comorbidities | 1) Number of healthcare barriers correlated with increased mental/somatic comorbidities, 2) Barriers correlated with autism severity, 3) Barriers related to unemployment, 4) Barriers associated with need for increased outpatient and emergency treatment, 5) Communication difficulties with providers, 6) Sensory issues in healthcare settings, 7) Problems in the waiting room, 8) Challenges with healthcare navigation, 9) Difficulties with healthcare logistics and appointments, 10) Executive functioning challenges impacting healthcare access | 1) Below-average mental health-related quality of life (1.5 standard deviations below norm), 2) Reduced physical health-related quality of life, 3) Higher rates of mental and somatic comorbidities, 4) 86.5% diagnosed with Asperger syndrome, 5) Average diagnosis at age 32.5 years (later diagnosis associated with poorer outcomes), 6) 44.4% employed, 55.6% unemployed, 7) Autistic adults with later diagnosis reported lower mental health-related quality of life, 8) High rates of healthcare barriers (almost double that reported in US studies) | No |
| Ahmed A. Mohamed, | (1) 81.9% society/family barriers impact seeking help, (2) 70.3% personal barriers prevent consultation, (3) 63.5% medical barriers discourage treatment, (4) 25.9% view psychiatric illness as shameful/stigmatizing, (5) 35.9% believe patients labeled as "crazy", (6) 34.3% find emotional expression difficult, (7) 39.5% fear reduced marriage prospects. | no | (1) Social stigma as main obstacle, (2) Shame when discussing mental health, (3) Cultural beliefs attributing illness to evil eye/envy, (4) Hospital admission fear, particularly in females, (5) Low confidence in medication effectiveness, (6) Gender differences: males concerned with social perception, females with treatment quality. | (1) Stigma preventing help-seeking, (2) Fear of negative labels, (3) Concerns about reduced marriage/employment opportunities, (4) Communication barriers about condition, (5) Internalized shame. | No |
| Fintan Sheerin, | 1) Healthcare providers reluctant to treat intellectual disability, 2) Resource limitations blocking service innovation, 3) Lack of pathways for age-related conditions, 4) Diagnostic overshadowing causing treatment delays, 5) 93.3% struggling with complex aging needs, 6) Only 10% of services fully meeting needs, 7) Per-capita funding approach fundamentally unsustainable, 8) Housing, staffing, and equipment all significantly impact service capability, 9) Increased health inequalities when aging with intellectual disabilities. | 1) Multidisciplinary coordination improving care quality, 2) Partnerships between disability/mainstream services supporting aging in place, 3) COVID-19 creating opportunities for individualized routines, 4) Some successful end-of-life care at home implementations, 5) Step-down facilities supporting transition back to community living, 6) Staff scheduling flexibility accommodating changing needs, 7) Specialized dementia accommodation where available. | 1) Static funding despite increasing care complexity, 2) Unsuitable housing for mobility/medical equipment needs, 3) Staff shortages in specialized intellectual disability nursing, 4) Siloed healthcare services preventing coordinated care, 5) Inflexible day services limiting retirement options, 6) Slow resource approval process during rapid health changes, 7) Regulatory requirements forcing relocation from preferred homes, 8) Difficulty adapting rental properties for accessibility, 9) Reliance on agency staff lacking intellectual disability experience, 10) "Congregated" funding limiting individualized service responses. | 1) Forced relocation when needs change, 2) Placement in generic nursing homes lacking specialized knowledge, 3) Loss of familiar environment and routines, 4) Reduced autonomy over daily schedule, 5) Individual preferences overlooked amid resource constraints, 6) Declining health forcing unwanted community participation to meet regulatory standards, 7) Limited retirement options unlike general population, 8) Needs increasing too rapidly for service adaptation, 9) Social impact on housemates when one person's needs intensify. | 1) Supplementing nursing home care due to staffing gaps, 2) Frustration with fragmented healthcare approach, 3) Persistent advocacy needed for proper diagnoses, 4) Ongoing support provision after institutional transition, 5) Trust issues with mainstream care quality, 6) Taking on night duty roles themselves in nursing homes, 7) Coordinating between disability services and nursing homes, 8) Epilepsy as unrecognized dementia precursor not communicated to families. |
| Laura Hughes-McCormack, | ) Healthcare inequalities gap reduced over time, 2) Management improved for adults with intellectual disabilities (OR=5.32), 3) No significant improvement for general population (OR=0.74), 4) Healthcare remained poorer compared to general population (OR=0.38 in 2014, improved from OR=0.05 in 2007-2010), 5) In 2014, healthcare comparable on 49/78 (62.8%) indicators, 6) Significant differences still existed on 29/78 (37.2%) indicators, 7) In 2007-2010, 46.4% of indicators had less than 50% achievement for intellectual disability population, 8) By 2014, only 12.8% had less than 50% achievement, 9) Improvements observed across 8 of 12 conditions measured. | 1) Health check programs for adults with intellectual disabilities, 2) Dedicated support from intellectual disabilities health services to primary care, 3) Community intellectual disabilities teams with specialized psychiatrists and nurses, 4) Coordinated care between primary care and specialist services, 5) Higher recording of certain health indicators (blood pressure, smoking status), 6) Better management of psychosis compared to general population, 7) Financial incentives for general practitioners to identify intellectual disability population. | 1) Diagnostic overshadowing and delays in diagnosis, 2) Lack of experience and training in managing intellectual disability patients, 3) Challenges in primary care management of specific conditions, 4) Poor health promotion participation, 5) Particularly low cervical screening rates (22.9% vs 90.7% in general population), 6) Depression management receiving less specialized support than psychosis, 7) Limited access to educational programs for diabetes, 8) Multiple chronic conditions requiring complex care coordination, 9) Geographically inconsistent service provision. | No | no |
| R.S. Balogh, | 1)Diabetes prevalence higher in IDD (16.0% vs 9.7%) 2)6-year consistent disparity 3)Higher incidence in IDD 4)Largest differences ages 30-39 5)Women with IDD: higher prevalence than men 6)2.6× preventable hospitalizations | [context] 1)Universal healthcare 2)Data surveillance 3)High-risk targeting 4)Earlier screening 5)Specialized programs 6)Risk recognition | 1)Poor diet, sedentary lifestyle 2)Cognitive limitations 3)Self-management difficulties 4)Medication side effects 5)Inadequate primary care 6)No targeted education 7)Limited provider awareness | [context] 1)Higher diabetes burden 2)Younger onset 3)More complications 4)Self-management challenges 5)Preventative care barriers 6)Medication complications | Experiences of caregivers: [From context, not explicitly stated] 1)Self-management support challenges 2)Need specialized education 3)Navigation difficulties 4)Increased monitoring needs 5)Cognitive adaptation requirements |
| Maji Hailemariam, | 1)Current absence of mental health services major barrier 2)Geographic distance significant challenge 3)Affordability primary on-going barrier 4)Transportation difficulties 5)Low awareness levels 6)Preference for traditional/religious healing 7)Financial challenges for long-term treatment | [ context] 1)Service integration into primary care 2)Treatment services within reach 3)Reduced transportation costs 4)Lower indirect costs 5)Financial support mechanisms 6)Community awareness raising 7)Innovative approaches like telephone consultations | [ context] 1)Service integration into primary care 2)Treatment services within reach 3)Reduced transportation costs 4)Lower indirect costs 5)Financial support mechanisms 6)Community awareness raising 7)Innovative approaches like telephone consultations | [ context] 1)Delayed help-seeking 2)Financial burden 3)Restraint/chaining due to lack of care 4)Traditional healing prioritized 5)Multiple indirect costs 6)Long-term care challenges 7)Declining social support over time | [ context] 1)Difficulty leveraging social support 2)Financial strain 3)Challenges with transportation 4)Conveying disturbed patients difficult 5)Seeking traditional healing first 6)Diminishing community support 7)Long-term caregiving burden |
| Melissa L. Desroches, | 1)Person-centered approach essential 2)Best practices organized into 9 guidelines (42 items) 3)Telehealth offers solutions to transportation barriers 4)Technology/device access critical 5)Support persons improve access success 6)Flexibility needed for individual needs 7)Communication adaptations necessary 8)Digital literacy affects access 9)Concerns about equity based on disability status | [ context] 1)Appropriate device access 2)High-speed internet 3)Support person availability 4)Tailored reminders 5)Clear communication accommodations 6)Training/practice sessions 7)Telehealth option for those with transportation barriers 8)Flexibility in communication methods | 1)Digital literacy challenges 2)Financial barriers to internet/devices 3)Privacy/security concerns 4)Technology operation difficulties 5)Need for support person 6)Lack of accommodations 7)Provider inexperience with developmental disabilities 8)Communication challenges 9)Insurance coverage limitations | [context] 1)Variable experiences with telehealth 2)Need for individualized approaches 3)Technology operation challenges 4)Communication barriers 5)Privacy concerns 6)Need for clear explanations 7)Importance of trust with provider 8)Value of choice between telehealth/in-person options | [context] 1)Managing technology for person 2)Balancing support without overriding autonomy 3)Facilitating communication 4)Providing emotional regulation support 5)Coordinating care 6)Need for education/training 7)Managing privacy concerns 8)Ensuring environment setup appropriate |
| Janet Golder, | 1)98% procedure completion rate 2)Three-tiered intervention model successful 3)Mild/moderate sedation sufficient for 67% of procedures 4)91% accurate sedation prediction 5)197 procedures completed including immunization (61%) and oral health (23%) 6)55% patients received secondary procedures 7)Living situation, procedure type and disability complexity affect sedation requirements | [ context] 1)Community-based service delivery 2)Individualized assessment approach 3)Tailored sedation options 4)Multiple procedure completion in single visit 5)Comprehensive pre-procedure planning 6)Person-centered care approach 7)Well-trained disability specialists 8)Post-procedure follow-up | 1)Uncertainty with healthcare environment 2)Communication difficulties 3)Diagnostic overshadowing 4)Health literacy issues 5)Fear and negative expectations 6)Decreased autonomy 7)Inadequate medication management 8)Affordability concerns 9)Lack of care coordination 10)Multiple disabilities complicating care | [context] 1)Need for personalized assessment 2)Tailored sedation requirements based on individual needs 3)Procedure anxieties requiring support 4)Multiple disability types increasing sedation needs 5)Successful procedure completion with appropriate support 6)Behavior concerns managed effectively 7)Living situation impacts procedure experience | [context] 1)Partnership with healthcare team essential 2)Involvement in assessment process 3)Support role during procedures 4)Coordination responsibilities 5)Managing behaviors before procedures 6)Providing information about previous experiences 7)Family members important for milder sedation requirements |
| Errol Cocks, | 1)Profound experiences of loss 2)Social barriers and isolation 3)Lack of meaningful occupation 4)Employment barriers 5)Limited family support 6)Fragmented service systems 7)Lack of specialized dual diagnosis services 8)Inappropriate accommodation 9)Financial constraints 10)Poor service integration | [context] 1)Family support 2)Meaningful occupation and activities 3)Person-centered approach 4)Specialist dual diagnosis services 5)Independent living options 6)Enhanced training for service providers 7)Collaborative service systems 8)Long-term support perspective 9)Strength-based approaches 10)Stable housing | 1)Lack of service integration 2)Limited expertise in dual diagnosis 3)No clear policy direction 4)Financial constraints 5)Transportation difficulties 6)Inappropriate medication management 7)Inadequate employment support 8)Unsuitable accommodation options 9)Limited social acceptance 10)Complex bureaucratic systems | 1)Loss of relationships and roles 2)Social isolation 3)Inability to engage in previous activities 4)Depression 5)Difficulty making independent choices 6)Social stigma 7)Lack of meaningful occupation 8)Shared accommodation challenges 9)Financial hardship 10)Medication issues | [context] 1)Adopting medical roles 2)Abandoning career plans 3)Feelings of helplessness 4)Managing medication 5)Monitoring behavior changes 6)Providing transportation 7)Limited respite opportunities 8)High energy demands 9)Emotional impact of suicidal statements 10)Isolation |
| Lisa A. Razzano, | 1)Significantly higher prevalence of 14 out of 17 medical conditions versus general population 2)Top conditions: hyperlipidemia (45%), hypertension (44%), asthma (28%), arthritis (22%), diabetes (21%) 3)Treatment rates below 70% for half of ongoing conditions 4)Mean 2.8 co-occurring conditions per person 5)87% had at least one medical condition 6)Racial/ethnic minorities twice as likely to have hypertension and diabetes 7)Women twice as likely to have diabetes 8)Schizophrenia diagnosis associated with lower hypertension and arthritis rates | [context] 1)Higher treatment rates for certain conditions (diabetes 94%, hypertension 89%) 2)Medicaid coverage 3)Non-minority status 4)Higher health self-efficacy 5)Gender-specific services 6)Support from healthcare providers 7)Community health screenings 8)Access to public health services | 1)Low treatment rates for many conditions (cancer 50%, stroke 42%) 2)Racial/ethnic minority status 3)Female gender for some conditions 4)Lower health self-efficacy 5)Poverty and material resource limitations 6)Lack of care coordination 7)Challenging living environments 8)Limited access to healthy food 9)Stressful environments 10)Multiple co-occurring conditions | [context] 1)High burden of co-occurring conditions 2)Medical vulnerability 3)Undertreatment of many conditions 4)Challenges with health self-management 5)Exposure to chronic stress 6)Unhealthy coping behaviors 7)Difficulty managing respiratory conditions 8)Cognitive impairments affecting health reporting 9)Multiple treatment needs 10)High smoking rates | [ context] 1)Need to coordinate multiple healthcare services 2)Support requirements for medical/psychiatric treatment 3)Challenges with medication management 4)Family responsibilities competing with healthcare 5)Need for transportation assistance 6)Supporting health self-efficacy 7)Addressing unhealthy coping mechanisms 8)Managing complex treatment regimens |
| Amy Oliver, | 1) decentralized fragmented healthcare system during COVID-19 affected service access, 2) digital exclusion prevented access to mental health support/treatments, 3) service users without technology couldn't access remote healthcare, 4) loan devices facilitated therapy participation and healthcare communication, 5) personal/household circumstances affected technology engagement, 6) digital inclusion increased healthcare system engagement, 7) improved control over healthcare appointment management. | 1) decentralized fragmented healthcare system during COVID-19 affected service access, 2) digital exclusion prevented access to mental health support/treatments, 3) service users without technology couldn't access remote healthcare, 4) loan devices facilitated therapy participation and healthcare communication, 5) personal/household circumstances affected technology engagement, 6) digital inclusion increased healthcare system engagement, 7) improved control over healthcare appointment management. | 1) lack of suitable devices/internet connectivity, 2) poor digital skills/confidence, 3) financial constraints, 4) lack of awareness about available support, 5) online security concerns, 6) disability/mental health affecting engagement, 7) reading/writing difficulties, 8) perceived age-related technology barriers, 9) concerns about damaging equipment, 10) limited understanding of loan terms. | 1) feeling digitally excluded/isolated, 2) forced to learn technology during COVID-19, 3) improved wellbeing through digital access, 4) greater sense of empowerment/control, 5) reduced social isolation, 6) ability to engage privately in therapy from home, 7) technology facilitating entertainment/distraction from symptoms, 8) concerns about becoming overly reliant on technology. | no |
| Kevin Lu, | 1) 41.9% respondents reported financial barriers to healthcare, 2) significant decreasing trend in barriers 2011-2017, 3) respondents with barriers 36% less likely to report good health status (OR 0.64), 4) 31% less likely to receive home healthcare (OR 0.69), 5) 33% more likely to have hospital stays (OR 1.33), 6) 50% more likely to have emergency department visits (OR 1.50). | 1) health insurance coverage, 2) increasing coverage rate (90.9% in 2011 to 95.5% in 2017), 3) Affordable Care Act implementation associated with decreased financial barriers, 4) higher family income, 5) higher education level associated with better health outcomes. | 1) unaffordability of medical care, 2) dental care costs, 3) eyeglasses costs, 4) mental healthcare costs, 5) follow-up care expenses, 6) specialist visit costs, 7) lower income (<$50,000), 8) lack of health insurance, 9) younger age (under 65), 10) non-white race/ethnicity. | 1) reduced likelihood of reporting good health status (36% less likely), 2) decreased access to preventive services, 3) reduced home healthcare visits, 4) increased reliance on emergency services, 5) increased hospitalization rates, 6) higher economic burden (85% higher odds), 7) delayed detection of cognitive impairment progression. | no |
| Sarah Wigham, | 1) primary care interfaces misaligned with needs of people with learning disabilities, 2) five themes identified: prioritization, proactivity, innovation/improvement, personalization, prevention/follow-up, 3) automated telephone systems create barriers, 4) incorrect coding/identification prevents access to appropriate care, 5) lack of reasonable adjustments, 6) difficulties following up with secondary/tertiary care. | 1) prioritizing people with learning disabilities, 2) thorough health checks with longer appointments, 3) accurate identification using registers/codes, 4) proactive outreach especially to unsupported individuals, 5) staff training and sharing good practice, 6) accessibility champions, 7) personalized flexible service, 8) continuity of care with same practitioners, 9) accessible information before/during appointments, 10) direct communication with the person. | 1) busy waiting rooms causing anxiety, 2) long waiting times, 3) automated telephone systems, 4) self-check-in technologies, 5) incorrect/missing diagnostic coding, 6) fear of health professionals from past experiences, 7) lack of awareness about health check entitlement, 8) atypical symptom presentation, 9) communication difficulties, 10) limited staff understanding of reasonable adjustments. | 1) feeling anxious in busy/noisy waiting rooms, 2) difficulty with automated systems, 3) unaware of health check entitlements, 4) valuing continuity with same healthcare professionals, 5) preferring "yearly" rather than "annual" terminology, 6) disliking being "talked over", 7) finding accessible information and pre-appointment preparation helpful, 8) appreciating picture-based questionnaires, 9) experiencing variable quality of health checks. | 1) difficulty identifying health issues in non-speaking individuals, 2) challenges with appointment timing and waiting, 3) struggling to explain disability-related needs to healthcare staff, 4) reporting "hit-and-miss" health check provision, 5) recognizing importance of reasonable adjustments, 6) needing to advocate for timely appointments, 7) carrying responsibility for coordinating follow-up care. |
| Michael Brown, | 1) cognitive limitations affect understanding of diabetes diagnosis/treatment, 2) unsuitable education programs for ID patients, 3) need for personalized accessible information, 4) limited time in clinic appointments, 5) need for joint clinics between ID/diabetes services, 6) need for proactive care coordination between services. | 1) person-centered diabetes management, 2) making reasonable adjustments to services, 3) tailored education programs, 4) joint clinics between ID and diabetes services, 5) longer appointment times, 6) continuity of care with same practitioners, 7) coordinated care between services, 8) additional support from ID practitioners, 9) adapted information resources, 10) designated diabetes nurse with ID interest. | 1) cognitive limitations affecting understanding, 2) lack of accessible information, 3) limited time in clinic appointments, 4) complexity of diabetes education programs, 5) limited engagement with self-management, 6) staff turnover in support services, 7) poor communication between services, 8) limited knowledge of diabetes among ID practitioners, 9) limited knowledge of ID needs among diabetes practitioners, 10) lack of care coordination between services. | no | no |
| Rafat Hussain, | 1) considerable multimorbidity (mean 3.8 conditions/person), 2) arthritis (40%), diabetes (26%), cardiovascular (23.6%), mental health (34.5%) most common, 3) significant barriers linked to socioeconomic disadvantage, 4) health conditions evolving from earlier life disadvantage, 5) limited preventative care. | : 1) reasonable access to primary care physicians, 2) specialist access for 50% of participants, 3) disability pension support, 4) community-based care enabling independent living, 5) health reviews and targeted screening. | 1) rural geographic barriers, 2) cumulative socioeconomic disadvantage, 3) limited health screening, 4) GPs' low confidence managing older people with ID, 5) inadequate healthcare provider training, 6) lack of preventative approaches. | 1) high multimorbidity (53.5% with 2-6 conditions), 2) significant mental health disorders (34.5%), 3) prevalent sensory impairments (44.5%), 4) higher condition rates among women, 5) different health patterns between younger-older (<65) and older-older (65+) adults. | no |
| Laura McKernan Ward, | 1) higher cancer mortality in ID population (SMR=1.20) despite lower/similar incidence, 2) metastatic cancer of unknown primary origin significantly higher (female SIR=1.70, male SIR=2.08), 3) excess mortality for colorectal (SMR=1.54), kidney (SMR=2.01), female genital (SMR=2.34), breast (female SMR=1.58), 4) late diagnosis indicated, 5) lower screening participation. | [context]1) tailored public health strategies, 2) carer awareness of cancer symptoms, 3) screening program support, 4) accessible health information, 5) clinician awareness of late presentation patterns in ID population. | [context]1) self-care challenges, 2) reliance on others to recognize symptoms, 3) communication barriers, 4) healthcare navigation difficulties, 5) lower screening uptake (45% less mammography), 6) healthcare professionals' inexperience with ID patients, 7) late presentation/diagnosis, 8) poorer treatment compliance. | [context]. 1) later cancer diagnosis, 2) poorer treatment outcomes, 3) higher mortality despite similar/lower incidence, 4) lower screening participation, 5) different cancer pattern compared to general population. | no |
| J.D. Reinhardt, | 1) prevalence of PTSD was 18.7% eight years post-earthquake, 2) participants perceived median of five environmental barriers, 3) most common barriers were lack of transportation and financial resources, 4) decreased physical/mental function associated with more environmental barriers, 5) more barriers significantly related to increased PTSD severity. | [context] The authors suggest but don't directly measure: 1) adequate rehabilitation services, 2) disability-inclusive environments, 3) integrated physical/mental health services, 4) improved insurance coverage, 5) disability pension/supports, 6) improved employment policies, 7) accessible transportation services. | 1) lack of long-distance transportation (most common barrier), 2) insufficient financial resources, 3) unfavorable climatic conditions, 4) lack of community-based rehabilitation, 5) limited insurance coverage, 6) insufficient disability pension, 7) inadequate employment policies, 8) limited rehabilitation capacity at community level, 9) absence of two-way referral system between hospital and community. | 1) physical/mental functioning significantly below population norms, 2) environmental barriers mediating relationship between functional limitations and PTSD severity, 3) experience of "two different types of psychological trauma" (initial earthquake trauma and ongoing disability-related trauma), 4) high prevalence of continued PTSD symptoms eight years post-earthquake. | no |
| Dan Qiu, | 1) contact coverage rates >80% for most services, 2) high utilization of follow-up services, 3) high utilization of physical examinations, 4) high utilization of medication distribution, 5) lower rates for inpatient/outpatient/rehabilitation services, 6) 56.8%-92.2% patients unaware of available services, 7) geographic differences between cities | 1) more professional resources in Guangzhou/Shenzhen, 2) higher mental health budgets in developed cities, 3) social medical assistance services, 4) free social medical insurance, 5) financial incentives for family caregivers | 1) policy participation restrictions, 2) poor service advertisement, 3) limited professional resources in less developed cities, 4) underfunded community mental health care, 5) migrants excluded by policy restrictions, 6) geographical accessibility challenges | 1) higher medication adherence with social assistance, 2) lower disability levels with rehabilitation services, 3) improved functioning with specialized services, 4) better health outcomes with service utilization, 5) different outcomes by service type | 1) higher guardianship grants coverage in Guangzhou/Shenzhen, 2) financial incentives for family care in some cities, 3) business insurance availability in some regions, 4) risk management policies for caregiver protection, 5) disparities in support between cities |
| E. Sally Rogers, | 1) improved continuity of care, 2) better community orientation of providers, 3) improved access to primary care with moderate exposure, 4) improved comprehensiveness of care, 5) better coordination of information and utilization with high exposure | 1) NP stationed in mental health setting, 2) integration with mental health team, 3) individualized healthcare planning, 4) promotion of wellness, 5) facilitation of specialty care access, 6) confidentiality barriers eliminated, 7) patient-centered approach | 1) low engagement despite initial interest, 2) lack of patient activation, 3) lack of sufficiently compelling health problems to seek care, 4) existing access to healthcare (Massachusetts context), 5) difficulty changing health perceptions/behaviors | 1) improved perceptions of primary care quality, 2) better access to primary care services, 3) improved care coordination and community orientation, 4) few improvements in perceived health status, 5) limited changes in lifestyle behaviors | no |
| Monika Mitra, | 1) 564 per 10,000 deliveries with severe maternal morbidity in women with IDD vs. 239 in women without, 2) 284 per 100,000 deliveries resulted in maternal mortality for women with IDD vs. 69 for women without, 3) higher risk of severe maternal morbidity after adjusting for sociodemographic characteristics (1.74 times) and obstetric comorbidity (1.23 times), 4) mortality risk remained 2.3 times higher after adjustments | [context] 1) higher rates of public insurance coverage for women with IDD (Medicaid 54.2%, Medicare 18.7%), 2) larger hospitals had higher utilization by women with IDD, 3) regional differences in access identified | [context]: 1) inadequate prenatal care, 2) difficulties communicating symptoms, 3) negative clinician attitudes, 4) provider misconceptions about sexual/reproductive needs, 5) delays in diagnosis/treatment, 6) poor patient-provider communication, 7) lack of accessible health information, 8) lower health literacy | 1) 2.4 times higher risk of severe maternal morbidity, 2) 4 times higher risk of maternal mortality, 3) greater prevalence of pre-existing chronic conditions, 4) higher rates of pregnancy complications, 5) higher obstetric comorbidity index scores | no |
| Marisa Brown, | 1) screening rates exceeded general population in 6 out of 7 categories, 2) highest screening rates for cholesterol (96.4%), prostate (94.5%, men), mammography (94.5%, women), hypertension (94.4%), 3) lowest rates for chlamydia/STD testing, HIV testing, skin cancer screening, and dementia screening, 4) exceeded Healthy People 2020 targets in 6 areas | [context]: 1) implementation of systematic health screening tool, 2) court monitoring and oversight, 3) clear guidelines for preventive screening by age/gender, 4) registered nurse involvement, 5) required documentation of screenings, 6) community-based residential services | [context]: 1) provider biases and assumptions about sexual activity, 2) limited information about reasons for refused screenings, 3) limited sexual health education, 4) insufficient attention to decision-making capacity, 5) challenges in monitoring non-institutional settings, 6) variability in disability service agency practices | 1) individuals with ID can receive preventive health screenings at rates higher than general population, 2) women received osteoporosis, glaucoma and dementia screenings at higher rates than men, 3) men received Hepatitis B and C screenings more frequently than women, 4) refusal rates were low (highest for osteoporosis at 2% and glaucoma at 1%) | no |
| Charlotte Emily Mott, | 1) Delayed diagnosis 2) Lower self-examination rates 3) LD patients 4x higher mortality 4) 5-year survival 90.8% vs 97.2% 5) 10-year survival 88.4% vs 96.8% 6) Health inequalities causing premature deaths [Based on stated study findings cited from Afshar et al] | 1) Annual health checks 2) Standardized templates 3) Longer appointments 4) Flexible scheduling 5) Easy-read materials 6) Patient passports 7) Individualized plans 8) Support networks 9) LD liaison nurses 10) Carer involvement | [context] 1) Failure reporting abnormalities 2) Late presentation 3) Consent challenges 4) Limited trial access 5) Patient disempowerment 6) Undervalued carers 7) Poor legislative understanding 8) Inadequate adaptations 9) Insufficient training 10) Communication difficulties | [context] 1) Late presentation despite independence 2) Limited information retention 3) Brief information recall 4) Disempowerment in consent 5) Limited autonomy 6) Symptom reporting difficulties 7) Need for family support | [context] 1) Sister as main carer 2) Identified health concerns 3) Contacted providers during treatment 4) Attended appointments 5) Involved in decision-making 6) Care expectations beyond capabilities 7) Opinions undervalued |
| Natasha Layton, | 1) Lack of knowledge about rehabilitation 2) Service navigation difficulties 3) Limited/fragmented services 4) Negative attitudes from health providers 5) Therapeutic nihilism towards dementia 6) Transport limitations 7) High costs 8) Exclusionary policies 9) Limited appointments with same provider 10) Rural service shortages | [Context] 1) Education resources on rehabilitation 2) Dementia navigators 3) Self-assessment apps 4) Peer support 5) Specialized rehabilitation programs 6) Adequate funding 7) Longer consultations 8) Education for health professionals 9) Training for aged care/disability staff 10) Strength-based approaches | 1) Lack of information about rehabilitation 2) Healthcare professionals' negative attitudes 3) Low provider knowledge 4) Therapeutic nihilism 5) Exclusionary policies 6) Complex navigation systems 7) Transport difficulties 8) High costs 9) Limited public services 10) Fragmented services | [Context] 1) Unaware of available therapies 2) Unable to find help despite searching 3) Services not aligned with needs 4) Feeling perceived as "tucked away" 5) Difficulty navigating complex systems 6) Need for value-based approaches 7) Need for ongoing advocate | [Context] 1) Required to provide beyond-capability care 2) Critical role identifying health concerns 3) Need to navigate complex systems 4) Needed as advocates 5) Opinions often undervalued 6) Key role in rehabilitation engagement 7) Need additional support |
| R Asaad Baksh, | 1) 37% less non-invasive respiratory support 2) 40% less intubation 3) 50% less ICU admission 4) 56% higher mortality risk 5) 1.44 times faster death rate 6) Longer hospital stays 7) More severe presentation 8) Higher oxygen requirements 9) Higher respiratory rates 10) Earlier death trajectory | [Context] 1) Demographic matching for comparisons 2) Recognition of clinical need 3) Oxygen therapy provision 4) Extended hospital stays 5) Clinical frailty recognition 6) Special needs protocols 7) ID-specific data collection 8) Earlier intervention recommendations 9) Enhanced monitoring 10) Prioritization guidance | 1) Fewer symptoms reported/recognized 2) Clinical frailty misinterpretation 3) Reduced non-invasive support 4) Lower intubation rates 5) Restricted ICU access 6) Potential treatment discrimination 7) Communication difficulties 8) Digital exclusion 9) Late presentation 10) Inappropriate care limitations | [Context] 1) Reduced subjective symptom reporting 2) More altered consciousness/confusion 3) Higher seizure rates 4) Aggressive disease progression 5) Earlier mortality 6) Extended hospitalization 7) Higher respiratory rates 8) Greater oxygen needs 9) Higher frailty scores 10) Late presentation | [Context] 1) Symptom recognition challenges 2) Hospitalization reluctance 3) Healthcare communication barriers 4) Digital information limitations 5) Restricted hospital involvement 6) Advocacy difficulties 7) Complex discharge planning |
| David Mason, | 1) 73.4% support autism health checks 2) 67.9% want checks for all autistic people 3) 67.3% prefer starting in childhood/teens 4) 61.4% favor annual checks 5) 50.7% prefer 15-30 minute appointments 6) 86.9% willing to provide health information beforehand 7) 86.2% willing to share adjustment needs 8) 54.6% prefer letter contact 9) Significant age/disability effects 10) Few sex differences | [Context] 1) Knowledgeable, familiar clinicians 2) Low-sensory environments 3) Advance information 4) Online booking 5) Flexible appointments 6) Staff autism training 7) Adequate discussion time 8) Personalized adjustments 9) Accessible information 10) Written appointment summaries | 1) Staff lacking autism knowledge 2) Sensory-overloading environments 3) Inflexible appointments 4) Unclear communication 5) Support gaps 6) Being "put on the spot" 7) Lengthy forms 8) Unconsented assessments 9) Condescending attitudes 10) Limited scheduling options | [Context] 1) Strong support for tailored checks 2) Willing to share advance information 3) Desire for appointment flexibility 4) Preference for familiar clinicians 5) Communication challenges 6) Environmental sensitivities 7) Need for preparation materials 8) Care continuity preference 9) Healthcare anxiety 10) Need for provider understanding | [Context] 1) Varying support needs 2) Provider training importance 3) Communication flexibility needs 4) Value of preparation information 5) Implementation challenges 6) Tailored appointment importance 7) Support for reasonable adjustments |
| Jill Bradshaw, | 1) 97% experienced day service reduction 2) By August 2021, only 63% returned to day services 3) 12% returned to respite care 4) 36% attending community activities 5) 52% receiving less support 6) 47% paying for services not received 7) 50% still had visit restrictions 8) 22% still shielding 9) 61% GP consultations by telephone 10) Only 29% received annual health checks | [Context]1) Opportunities for social contact 2) Access to community activities 3) Opening day centers with increased funding 4) Better access to health services 5) Contact with allied health professionals 6) Trained staff 7) Consistent staff team 8) Staff creativity 9) Imaginative support 10) Digital access and support | 1) Closure of day centers 2) Visitor restrictions 3) Lack of specialized health services 4) Telephone consultations replacing in-person visits 5) Staff shortages 6) Untrained staff 7) Excessive shielding 8) Service reduction without alternatives 9) Shift from rights to protection focus 10) Risk-averse providers | [Context] 1) Increased social isolation 2) Mental health deterioration 3) Physical health decline 4) Increased behavioral distress 5) Loss of life skills 6) Reduced mobility 7) Depression 8) Less interest in activities 9) Loss of confidence 10) Increased anxiety/frustration | [Context] 1) Increased care responsibilities 2) Limited decision-making involvement 3) Anxiety about hospital visits 4) Concerns about long-term skill loss 5) Financial impact 6) Physical/mental health impacts 7) Balance between infection protection and quality of life 8) Staff retention concerns 9) Concerns about culture shift 10) Ongoing care burden |
| Dr Jean-Francois Trani, | 1) Health care availability worsened (69% in 2005 vs 44% in 2013) 2) Positive experience with coverage declined (78% in 2005 vs 45% in 2013) 3) Village remoteness increased 4) Longer travel time to facilities (64.3 minutes in 2005 vs 84.4 minutes in 2013) 5) Reduced road connectivity 6) Formal education associated with better access 7) Higher asset levels associated with better access 8) Employment associated with better experience 9) Village remoteness negatively affected access 10) No improvement for people in remote areas | [Context] 1) Formal education 2) Higher economic status 3) Being employed 4) Village connectivity by paved road 5) Shorter time to reach health facility 6) Contract-based service delivery 7) Multidisciplinary team approach 8) Community health workers 9) Local recruitment 10) Performance-based funding | 1) Increased village remoteness 2) Lack of paved roads 3) Longer travel times 4) Lower socioeconomic status 5) Lack of education 6) Insufficient resources allocated to disability 7) Social stigma toward disability 8) Attitudinal barriers 9) Security issues 10) Lack of trained staff for rehabilitation | [Context] 1) Worsening perception of healthcare availability 2) Declining satisfaction with healthcare coverage 3) Inadequate services for specific needs 4) Economic barriers to access 5) Physical accessibility challenges 6) Communication difficulties with providers 7) Poor quality services 8) Preference for private healthcare 9) Long waiting times 10) Discrimination in healthcare settings | [Context] 1) Increasing care responsibilities 2) Greater financial burden 3) Limited support services 4) Difficulties accompanying patients 5) Communication challenges with providers 6) Transportation difficulties 7) Security concerns when traveling 8) Long waiting times 9) Lack of involvement in decision-making 10) Limited caregiver support |
| Veronika Reichenberger | 1) Good health literacy despite communication barriers 2) Community health agents as first contact 3) Inaccessible transportation 4) Poor urban infrastructure 5) Variable facility accessibility 6) High medication costs 7) Communication barriers 8) Inaccessible equipment 9) Limited home visits 10) Dependency on support | [Context] 1) Community health agents 2) "Atende+" transport service 3) Family support 4) Staff assistance 5) Home visits 6) Health literacy 7) Prior healthcare experiences 8) Social support networks 9) Accessible facilities 10) Free SUS services | 1) Communication barriers 2) Lack of sign language knowledge 3) Inaccessible transportation 4) Poor infrastructure 5) Variable facility accessibility 6) High medication costs 7) Insufficient home visits 8) Long waiting times 9) Brief consultations 10) Inaccessible equipment | [Context] 1) Dependency on logistical support 2) Self-medication 3) Waiting room challenges 4) Being ignored by providers 5) Missed examinations 6) Good health knowledge 7) Catastrophic expenses 8) Inaccessible booking systems 9) Transportation challenges 10) Reliance on private transport | [Context] 1) Accompanying to appointments 2) Providing transportation 3) Supporting communication 4) Managing appointments 5) Assisting with navigation 6) Medication management 7) Handling waiting room challenges 8) Managing patient anxiety 9) Supporting healthcare decisions 10) Coordinating comprehensive care |
| Carrie E. Henning-Smith, | 1) Higher delayed care with disabilities 2) Living alone increases barriers 3) Cost barriers highest for those alone 4) Transportation barriers higher with disabilities 5) Living alone with disability most disadvantaged | [Context] 1) Spouse presence 2) Higher income 3) Education 4) Insurance 5) Medicare Advantage 6) Family caregivers 7) Transportation 8) Care coordination 9) Flexible scheduling 10) Navigation support | 1) Cost 2) Transportation 3) Scheduling 4) Living alone with disability 5) Non-spousal households 6) Low income 7) Poor health 8) Caregiver time constraints 9) Service fragmentation 10) Coverage gaps | [Context] 1) Higher delay rates 2) Transportation challenges 3) Resource constraints 4) Cost barriers 5) Vulnerability when alone 6) Complex needs 7) Time-intensive care 8) Special transport needs 9) Scheduling problems 10) Navigation difficulties | [Context] 1) Time constraints 2) Competing duties 3) Transportation challenges 4) Coordination burden 5) Variable effectiveness 6) Non-spousal limitations 7) Scheduling needs 8) Respite requirements 9) Multiple roles 10) Navigation responsibilities |
| Stephanie E Rogers, | 1) 18.6% experienced healthcare discrimination (12.6% infrequently, 5.9% frequently) 2) 28.5% with frequent discrimination developed disability vs 14.7% without 3) 63% higher disability risk after adjustment 4) Age (28%), gender (12%), financial status (12%) most common discrimination reasons 5) Healthcare discrimination uniquely linked to disability | [Context] 1) Higher education 2) Higher income 3) Better health at baseline 4) White race 5) Married status 6) Proper patient communication 7) Preventive care use 8) Provider awareness 9) Respectful treatment 10) Provider education | 1) Frequent healthcare discrimination 2) Poor service/treatment 3) Provider communication issues 4) Age discrimination 5) Socioeconomic discrimination 6) Gender discrimination 7) Racial discrimination 8) Depression 9) Chronic conditions 10) Low healthcare system trust | [Context] 1) Higher discrimination rates 2) Poorer service 3) Communication barriers 4) Treatment delays 5) Prescription filling delays 6) Fewer preventive services 7) Less informativeness 8) Functional decline 9) Accessibility issues 10) Less psychosocial communication | No |
| Nancy A. Miller, | 1) Minorities less likely to have usual care source 2) Minorities more likely to have suboptimal care source 3) SES effects stronger for middle-aged adults 4) Black middle-aged adults had higher ED use 5) Income effects significant across multiple measures | 1) Higher income 2) Higher education 3) Having health insurance 4) Medicare/Medicaid coverage 5) Dual coverage 6) Having usual source of care 7) Living in metropolitan areas 8) Being White 9) Being older 10) Having private insurance | 1) Minority status 2) Lower income 3) Lower education 4) Lack of insurance 5) Having suboptimal usual care source 6) Physical accessibility issues 7) Provider cultural competency limitations 8) Being middle-aged 9) Complex disabilities 10) Environmental barriers | [contex] 1) Higher unmet healthcare needs 2) Difficulties obtaining usual source of care 3) More ED use with complex disabilities 4) Transportation challenges 5) Less preventive counseling 6) Higher smoking rates 7) Higher obesity rates 8) Exercise limitations 9) Secondary health conditions 10) Cultural barriers | No |
| Mohammad Hifz Ur Rahman, | 1) HSR lower among disabled/severely disabled older adults (except Ghana), 2) disparities persist after controlling for socioeconomic factors, 3) greatest disparity in South Africa (8-point difference), 4) Mexico highest HSR score (61), 5) South Africa lowest HSR score (50) | 1) Higher education, 2) increased wealth, 3) better amenities, 4) rural residence in Russia, 5) urban residence in India, 6) Mexico's inclusive disability policies | 1) Disability status, 2) lower socioeconomic status, 3) widowed status, 4) lack of skilled providers, 5) improper referral systems, 6) negative attitudes, 7) poor client orientation | 1) Limited decision-making involvement, 2) fewer care queries, 3) difficulty understanding medical information, 4) 4x poorer HSR reports, 5) service use disruption, 6) decreased system trust | No |
| Hussain Zandam | 1) Disabled reported poorer health system accessibility (0.72 vs 0.52), 2) experienced more barriers across all dimensions, 3) accommodation showed widest gap (0.68 vs 0.24), 4) disability remained significant after controlling for other variables, 5) accessibility and affordability were strongest predictors | 1) Higher SES, 2) urban location, 3) higher education, 4) employment, 5) higher income, 6) better self-reported health | 1) Physical accessibility issues, 2) affordability constraints, 3) rural location, 4) poor service accommodation, 5) limited knowledge of services, 6) availability barriers | 1) Worse socioeconomic factors, 2) poorer self-reported health, 3) higher rural residence, 4) greater use of traditional remedies, 5) multiple dimensional barriers | No |
| Sara H Rotenberg | 1) Small differences in antenatal care attendance, 2) No differences in provider types, 3) Higher doctor attendance at births (aOR=1.52), 4) High antenatal coverage overall (95.4%), 5) Nurses most common providers for both groups, 6) Small disability sample (3.1%), 7) No care quality data. | 1) Task-shifting to nurses/midwives, 2) Community health worker options, 3) Potential appropriate risk management through doctors, 4) Possible facility-based births, 5) Generally accessible services, 6) No disability-specific facilitators found. | 1) Small sample limiting analysis, 2) Transportation, provider attitudes, health information not measured, 3) Severe disabilities likely undercounted, 4) No quality/acceptability data, 5) Financial burden unmeasured, 6) Limited disability disaggregation, 7) Psychosocial/intellectual disabilities underrepresented. | 1) Similar antenatal attendance patterns, 2) Comparable provider types, 3) More doctor-attended births, 4) No data on care quality, preferences, autonomy, outcomes, 5) Severe disabilities likely excluded. | Not reported |
| Ali Lakhani | 1) Fewer inaccessible services post-lockdown. 2) More health conditions after restrictions. 3) Better outdoor autonomy. 4) Improved specialist access. | 1) Lifting restrictions. 2) Clear care definitions. 3) Telehealth. | 1) Distancing restrictions. 2) Unclear eligibility. 3) Face-to-face requirements. 4) Provider knowledge gaps. | 1) Limited outdoor autonomy during lockdown. 2) Increased shoulder/sexual issues post-lockdown. 3) Better service access. 4) Slight QOL improvement. | Not reported |
| Ana Oña, | pro-rich health inequalities 2) better health in high-income groups 3) 18% reported unmet needs 4) unmet needs explain health inequalities 5) Norway: lowest unmet needs (9%) 6) Morocco: highest (62%) | Not reported | 1)healthcare costs (Morocco 80%, China 52%) 2) unavailable services (Italy, Spain >30%) 3) transportation problems (South Africa 70%) 4) poor treatment (China 34%) 5) work conflicts (Japan 49%) 6) inadequate providers (France, Netherlands >45%) 7) information gaps (China 30%) | 1)better health in high-income groups 2) Switzerland/Japan: 22 years with injury 3) China/Brazil: <6 years with injury 4) South Korea: highest comorbidities 5) Brazil: lowest comorbidities | Not reported |
| Claire Ryan, | 1) Social e-Health users 3× more likely to e-communicate with providers,2) comorbidities increased e-communication likelihood (OR=1.64),3) higher education increased e-communication (OR=1.83),4)no variation by age, race, gender, or communication modality. | 1)social network health engagement,2) college education,3) multiple health conditions,4)health discussion social support network. | 1)healthcare communication barriers,2) limited ASL health information,3) lower education,4)insufficient health discussion networks. | 1)barriers to health information/services,2)provider communication challenges,3) poorer health outcomes,4)online platforms bypass barriers,5)social media enables ASL health information sharing. | Not reported |
| Bougangue Bassoumah, | 1) Lower clinic attendance. 2) Fewer facility deliveries (6/20 vs 15/20 non-disabled). 3) Reduced postnatal care within 48-hours. 4) Higher maternal/neonatal complication risks. | 1) Children assisting mobility/communication. 2) Marriage/children increasing social status. 3) Supportive healthcare professionals. 4) Government financial assistance. 5) Education (physical disabilities). | 1) Limited transportation. 2) Unemployment/poverty. 3) Cultural stigma. 4) Multi-level discrimination. 5) Inaccessible health information. 6) Disabled spouses. 7) Non-inclusive facilities. 8) Inadequate professional training. 9) Low social status. | 1) Verbal abuse. 2) Healthcare stigmatization. 3) Decision-making exclusion. 4) Family disrespect. 5) Information barriers. 6) Employment discrimination. 7) Confidence issues. 8) Inadequate pregnancy support. 9) Infant mortality. | 1) Mixed willingness to assist. 2) Poor health information relay. 3) Negative cultural attitudes. 4) Work prioritized over caregiving. |
| Jihyun Kwon, | 1) Lower MM diagnosis rate in disabled population (29.1 vs 39.4 per 100,000), 2) Higher dialysis treatment at diagnosis (16.3% vs 10.0%), 3) Lower autologous stem cell transplantation rates (37.5% vs 43.7%), 4) Shorter survival (36.8 vs 51.2 months), 5) Greater disparities for intellectual/psychological disabilities | 1) Medical aid program providing free basic services to lowest income bracket | 1) Low socioeconomic status, 2) Difficulty recognizing/expressing symptoms, 3) Physical mobility restrictions, 4) Caregiver dependence affecting treatment decisions, 5) Informed consent challenges, 6) Comorbidities masking symptoms | 1) Later-stage diagnosis, 2) Less intensive treatment options, 3) Poorer survival outcomes, 4) Higher complication rates at diagnosis | Not reported |
| Reuben Kalavina, | 1) Limited national rehabilitation services. 2) Single rehabilitation center nationwide. 3) Admission delays. 4) Intervention delays worsening outcomes. 5) Medication scarcity. | 1) Family/friend support. 2) Financial resources. | 1) Insufficient rehabilitation space. 2) High transport costs. 3) Post-stroke financial hardship. 4) Drug shortages. 5) Expensive private healthcare. 6) Few community rehabilitation resources. 7) Poor facility conditions. | 1) Mobility/function loss. 2) Self-care inability. 3) Dependency. 4) Income loss. 5) Social isolation. 6) Medication access difficulties. 7) Cognitive/emotional challenges. | 1) Care burden. 2) Role transformation. 3) Managing incontinence/speech/anger issues. 4) Employment disruption. 5) Social life reduction. 6) Gender disparity in burden. 7) Emotional distress. |
| Fareha Nishat, | 1) Most received adequate care 2) Physical disabilities: increased intensive care 3) Sensory disabilities: increased no/inadequate/intensive care 4) Intellectual/developmental disabilities: increased no/inadequate care 5) Multiple disabilities: increased inadequate/intensive care | 1) Universal healthcare 2) Chronic conditions 3) Pre-existing healthcare connection 4) Higher perceived risk 5) Primary care continuity | 1) Provider attitudes 2) No tailored resources 3) Communication barriers 4) Socioeconomic factors 5) Young age 6) Social disadvantage 7) Child services fears 8) Limited sexual health education 9) Late pregnancy recognition | 1) Intellectual/developmental disabilities most vulnerable 2) Some received excessive care 3) Delayed care entry 4) Fewer visits than recommended 5) Autonomy/dignity issues | Not reported |
| Carmen E Capo-Lugo, | 1) 22.4% no therapy services, 2) 30.5% consultation-only, 3) 47.1% consultation+intervention, 4) hemorrhagic stroke decreased consultation (OR=0.34 intracerebral, OR=0.52 subarachnoid), 5) severe stroke decreased consultation (OR=0.34), 6) moderate stroke increased interventions (OR=1.43), 7) pre-stroke ambulation increased interventions (OR=5.08) | 1) longer stays increased therapy access, 2) moderate stroke severity increased therapy, 3) pre-stroke ambulation increased interventions | 1) severe stroke, 2) hemorrhagic stroke, 3) pre-stroke disability, 4) procedures/tests (26.4%), 5) patient refusal (24.9%), 6) medical acuity (18.4%), 7) bed rest (10.9%), 8) weekend admission, 9) short hospitalization | Not reported | Not reported |
| Kavita Makan, | (1) 10.8-year diagnostic delay. (2) 3.4 mean healthcare visits pre-diagnosis. (3) 87% active disease despite treatment. (4) 69.9% psychological distress. (5) Two-thirds with functional limitations. | (1) Rheumatologist diagnosis (77.9%). (2) Higher socioeconomic status. (3) Private insurance access to biologics (42.5%). | (1) Few rheumatologists (115 for 56 million). (2) Limited MRI access (34.8%). (3) High private healthcare costs. (4) axSpA excluded from insurance minimum benefits. (5) Geographic specialist disparities. | (1) Limitations in physical exercise (68.5%). (2) Housework difficulties (59.6%). (3) Impact on social life (71.2% abandoned activities). (4) Impact on relationships (75.3% guilt feelings). (5) Female gender associated with worse outcomes. | Not reported |
| Robert Kokou Dowou, | 1) Three coping strategies: emotion-oriented, task-oriented, avoidance-oriented. 2) Family caregivers essential for management. 3) Religious activities primary coping mechanism. 4) Creative arts used as distraction techniques. 5) Social activities improved emotional well-being. | 1) Family support. 2) Religious community. 3) Health professional guidance. 4) Social engagement. 5) Caregiver transportation assistance. | 1) Financial constraints. 2) Inadequate family support. 3) Poor healthcare worker attitudes. 4) Long waiting times. 5) Drug unavailability. 6) Patient non-adherence. 7) Limited health insurance coverage. | 1) Religious faith as emotional support. 2) Media distraction techniques. 3) Social activities for diversion. 4) Self-motivation through condition acceptance. 5) Reported condition improvement with support. | 1) Provided medication, hygiene, transportation assistance. 2) Reported feeling overburdened. 3) Experienced financial strain. 4) Faced healthcare system delays. 5) Struggled with patient resistance to lifestyle changes. |
| Barbara Kołłątaj, | 1) 30.1% no equipment needs, 2) 9.8% unmet equipment needs, 3) 22.4% partially met needs, 4) 37.7% fully met needs, 5) 59.6% no home adjustment needs, 6) 15.9% unmet home adjustment needs, 7) 24.5% met home adjustment needs | 1) residential home living, 2) higher economic status, 3) older age, 4) urban residence | 1) poverty, 2) independent single living, 3) rural residence, 4) younger age, 5) injury/congenital disability | 1) highest equipment needs: walking aids, exercise equipment, dental prostheses, 2) highest home adjustment needs: bathroom modifications, doorstep removal, rails, 3) partial needs satisfaction linked to worse health | Not reported |
| Rajeswaran Thiagesan, | 1) Inaccessible healthcare facilities, 2) Irregular medication supply, 3) Unaffordable medications, 4) Inaccessible laboratories, 5) Insufficient counseling | 1) Disability pensions, 2) Free mobility aids, 3) Doorstep medicine delivery, 4) Community health volunteers | 1) Physical inaccessibility, 2) High costs, 3) Transportation difficulties, 4) Distance, 5) Medication unavailability, 6) Provider insensitivity, 7) Poor communication, 8) Lack of disability-specific guidelines | 1) Nutritious food inaccessibility, 2) Physical activity limitations, 3) Medication confusion, 4) Irregular monitoring, 5) Chronic pain, 6) Mental health issues, 7) Healthcare discrimination, 8) Poor quality of life | 1) Physical support challenges, 2) Communication difficulties, 3) Daily care burden, 4) Managing emotional responses |
| Elise‐Marie Dilger, | 1) MADs enable participation, 2) MADs enhance efficacy/independence, 3) Environmental-personal factor interaction affects participation quality, 4) Contextual factors determine participation experience. | 1) MADs, 2) Emotional support, 3) Barrier-free design, 4) Accessible facilities, 5) Adaptive workplaces, 6) Efficient device provision, 7) Supportive attitudes, 8) Personal acceptance. | 1) Architectural obstacles, 2) Inaccessible transportation, 3) Non-integrated accessibility, 4) Restrictive regulations, 5) Weather limitations, 6) Discrimination, 7) Information gaps, 8) Isolating accommodations. | 1) Ambivalence toward assistance, 2) Device-related dependencies, 3) Otherness perception, 4) Accessibility uncertainty, 5) Challenging acceptance process, 6) Activity withdrawal from barrier fatigue, 7) Social isolation from physical separation. | Not reported |
| Olena Bychkovska, | 1) 17% reported unmet healthcare needs, 2) highest in Morocco (62%), lowest in Switzerland/Spain (7%), 3) unavailability of services most common barrier (9%), 4) country of residence most significant factor | 1) higher income, 2) better health status, 3) residence in countries with better healthcare infrastructure, 4) higher government health expenditure, 5) lower out-of-pocket expenses | 1) service unavailability (9%), 2) unaffordability (7%), 3) service inappropriateness (4%), 4) service unapproachability (4%), 5) low income, 6) multiple comorbidities, 7) lower functional status | 1) more access difficulties than general population, 2) worse access with low income/poor health, 3) significant country-dependent variations | Not reported |
| Rosemary B Hughes, | 1) Internet primary for general health info, 2) providers primary for sexual/reproductive info despite negative experiences, 3) peer social media valued for lived experience, 4) info pathways vary by health topic, 5) limited disability-specific information availability | 1) self-advocacy, 2) peer social media support, 3) proactive information seeking, 4) finding disability-competent providers, 5) physical therapists for pelvic health information | 1) provider knowledge gaps, 2) physical inaccessibility, 3) provider negative attitudes, 4) rural/technology limitations, 5) medical issues inappropriately attributed to disability | 1) provider dismissal, 2) shock reactions to sexual health inquiries, 3) inadequate rehabilitation information, 4) burden of educating providers, 5) reliance on peer support over healthcare system | Not reported |
| Michelle M. Y. Wong, | 1) Disrupted recovery trajectory. 2) Postponed physical assessments. 3) Reduced daily care support. 4) Limited facility access. 5) Decreased provider contact. | 1) Efficient telehealth. 2) Visual connection via video. 3) Eliminated transportation needs. 4) Lower-energy appointment preparation. 5) Walking as accessible exercise. | 1) Staff shortages. 2) COVID exposure fears. 3) Equipment costs. 4) Rural connectivity issues. 5) Cognitive/physical limitations for virtual participation. 6) Absence of physical examinations. 7) Environmental restrictions. 8) Inadequate financial support. | 1) Feeling "forgotten" by policymakers. 2) Recovery stagnation. 3) Increased fatigue. 4) Social isolation. 5) Worsening pain. 6) Developing coping mechanisms. 7) Valuing provider interactions. 8) Missing in-person connections. 9) Experiencing stigma. | Not reported |
| Seon Mee Park, | 1) lower diagnostic rates in severe disabilities (110.6 vs 136.5 per 100,000), 2) earlier diagnosis age but reduced surgery access (aOR=0.52), 3) decreased chemotherapy utilization (aOR=0.76), 4) increased mortality (aHR=1.05), 5) worst outcomes for mental/brain disabilities | 1) universal insurance, 2) 5% coinsurance, 3) $1000 copay maximum, 4) national disability registration | 1) severe disability, 2) mental/brain impairments, 3) poverty, 4) rural residence, 5) communication challenges, 6) female gender with mental disability | 1) younger diagnosis age, 2) higher comorbidities, 3) incomplete staging, 4) less intensive treatment, 5) poorer survival | Not reported |
| Tare Lowe, | 1) Limited hearing loss education 2) Services with Eurocentric design 3) Delayed help-seeking 4) Lack of cultural assessments 5) Māori underrepresentation in services | 1) Te reo hearing assessments 2) Māori workforce representation 3) Co-designed services 4) Cultural relationship-building 5) Integration of Māori values 6) Screenings in Māori health services | 1) Cost 2) Discrimination fear 3) Unwelcoming environments 4) Cultural safety absence 5) Limited Māori workforce 6) Healthcare anxiety 7) Biomedical approach misalignment | 1) "Not for me" perception 2) Delayed help-seeking 3) Unwelcoming spaces 4) Inequitable treatment fear 5) Preference for Māori providers 6) Desire for cultural representation | 1) Cost concerns 2) Observed help-seeking delays 3) Desire for culturally appropriate services |
| Niu Tian, | 1) Lower uninsurance in active epilepsy (6.5% vs 11.0%). 2) Less private insurance, more Medicaid in epilepsy patients. 3) Higher medical bill problems (27.9% active, 27.6% inactive vs 14.0%). 4) Medicare potentially reducing financial barriers for older epilepsy patients. | 1) Higher insurance coverage. 2) Medicare for seniors reducing costs. 3) Medicaid expansion benefiting low-income chronically ill. 4) Epilepsy Foundation Helpline connecting to resources. | 1) Transportation difficulties (6.9% active vs 1.7% without). 2) Provider availability (5.4% active vs 3.1%). 3) Specialty care affordability (7.5% active vs 4.1%). 4) Mental healthcare costs. 5) Medication rationing to save money. 6) Dental/vision care unaffordability. | 1) Medical bill problems, worse below poverty level. 2) Higher public insurance dependency. 3) Prescription affordability issues. 4) Transportation barriers delaying care. | Not reported |
| Dikaios Sakellariou, | 1) Lack of professional/institutional preparation for disability needs. 2) Non-acknowledgment of disability-specific requirements. 3) "Unseeing" disability during cancer treatment planning. 4) Widespread physical inaccessibility of facilities. | 1) Flexible service provision. 2) Professionals considering specific needs. 3) Adaptable procedures accommodating discomfort. | 1) Poor communication between specialists. 2) Discontinuity of care. 3) Unacknowledged disability needs. 4) Transportation barriers. 5) Inadequate parking. 6) Inaccessible buildings/equipment. 7) Inflexible appointments. 8) Limited disability awareness among professionals. | 1) Disability ignored/"unseen". 2) Self-advocacy necessity. 3) Personal expertise disregarded. 4) Navigation anxiety. 5) Equipment inadequacy. 6) Inappropriate procedures. 7) Dependency on others for access. | 1) Expertise ignored. 2) Work disruption. 3) Forced advocacy role. |
| Verusia Chetty | 1) hospital-based services inaccessible; 2) community-based rehabilitation promising alternative; 3) framework needed integrating policy, patient-centered care, multidisciplinary approaches; 4) potential for trained community workers to deliver local services. | 1) trained community healthcare workers; 2) student placement from local institutions; 3) home-based care; 4) efficient communication; 5) strong leadership; 6) stakeholder collaboration; 7) patient/caregiver education. | 1) centralized services; 2) transportation challenges; 3) financial constraints; 4) difficult terrain; 5) staff shortages; 6) high patient volume; 7) poor multidisciplinary collaboration; 8) inadequate discharge planning; 9) knowledge gaps; 10) lack of follow-up. | 1) physical inability to reach services; 2) financial barriers; 3) disability-poverty cycle; 4) disrupted care continuity; 5) poor community reintegration. | 1) full-time care preventing employment; 2) financial constraints; 3) knowledge gaps about rehabilitation; 4) difficulty transporting dependents to facilities. |
| Amber Willink, | 1) 87% untreated hearing trouble 2) women/minorities/low-income less likely using hearing aids 3) main touchpoints: pharmacies (64%), medical providers (50%) 4) 20% accessed neither 5) clinic-based model inadequate | 1) pharmacy-based delivery reaching vulnerable populations 2) leveraging medical provider visits 3) online models for internet users 4) community paraprofessionals 5) retail integration | 1) high cost 2) no insurance coverage 3) stigma 4) transportation 5) mobility issues 6) system complexity 7) lack of provider recommendations 8) time-consuming process | Not reported | Not reported |
| Whitney Powell, | 1) Impacts communication, relationships, 2) reduces medical compliance, 3) impairs work performance, 4) compromises safety, 5) causes social isolation | 1) Primary care referrals, 2) VA referrals, 3) employment-required screenings, 4) willingness if affordable | 1) Provider scarcity in rural areas, 2) long travel distances, 3) prohibitive costs, 4) no insurance coverage, 5) limited awareness, 6) inconvenient hours, 7) quality concerns | 1) Communication difficulties, 2) embarrassment, 3) isolation, 4) workplace challenges, 5) phone communication problems, 6) group anxiety, 7) safety risks | 1) Repetition frustration, 2) increased worry, 3) relationship strain, 4) skepticism about severity, 5) treatment non-adherence concerns |
| Mansha Mirza, | 1)Professional interpreter use limited by time/resources, 2) environment impacts interpretation method feasibility, 3) clinicians prefer professional interpreters for complex interactions, 4) patients value expertise over language ability, 5) low interpreter requests from patients. | 1)In-person interpretation, 2) rehabilitation-specific terminology knowledge, 3) clinicians' basic language efforts, 4) cultural therapy adaptations, 5) mobile technology for dynamic sessions, 6) interpreter continuity. | 1)Limited professional interpreters, 2) budget constraints, 3) environmental noise affecting remote interpretation, 4) privacy issues, 5) inadequate clinician training, 6) extended session times, 7) untranslated assessments. | 1)Low service expectations, 2) appreciation despite barriers, 3) expertise prioritized over language, 4) concerns about interpreter wait times, 5) inaccurate video interpretation, 6) valued clinicians' language attempts. | Not reported |
| Ruth Bailey, | 1) Barriers create embodied well-being threats, 2) Physical/emotional distress, 3) Disempowering negotiations, 4) Barriers from design, practices, power inequities | 1) Collaborative relationships with staff, 2) Recognition of embodied difference, 3) Transport personnel support, 4) Advance equipment checks | 1) Large hospital distances, 2) Equipment unavailability, 3) Non-inclusive medical equipment, 4) Rigid protocols, 5) Staff disability awareness gaps, 6) Inaccessible toilets, 7) Inflexible appointments, 8) Power imbalances | 1) Responsibility without power, 2) Anticipatory stress, 3) Competing threats during decision-making, 4) Stigmatization fears, 5) Feelings of exclusion, 6) Emotional labor burden | Not reported |
| Sarah E. Bauer, | 1) physical environment barriers (OR=16.6) 2) clinical experience barriers (OR=13.9) 3) communication barriers (OR=6.7) 4) care coordination difficulties (OR=5.7) | Not reported | 1) transportation (12% vs 1%) 2) facility entry (8% vs 0.5%) 3) exam table access (20% vs 1%) 4) provider knowledge deficit (14% vs 2.4%) 5) care coordination (16% vs 3%) 6) inaccessible facilities/equipment 7) poor communication | 1) provider communication challenges 2) medical knowledge gaps 3) physical accessibility issues 4) transportation barriers 5) care coordination problems | Not reported in the study. |
| Kevin J. Bennett, | 1) Higher diabetes prevalence with disabilities (12.1% vs 7.1%), 2) Low service receipt both groups, 3) Foot exams higher with disabilities, 4) No disparities other services, 5) <30% received eye exams, 6) 40% received no services, 7) <4% received all services | 1) Usual source of care, 2) Age 65+, 3) Higher income, 4) Multiple chronic conditions | 1) Uninsured status, 2) Hispanic ethnicity, 3) Lower education, 4) Rural residence, 5) Poor care coordination | Not reported | Not reported |
| Neelam Borade, | 1) Limited suitable device availability, 2) Certification delays, 3) Financial barriers, 4) Awareness gaps | 1) Limited suitable device availability, 2) Certification delays, 3) Financial barriers, 4) Awareness gaps | 1) Healthcare provider apathy, 2) High costs, 3) Inaccessible infrastructure, 4) Transport difficulties, 5) Limited quality devices, 6) Inadequate repair services, 7) Stigma | 1) Greater independence, 2) Reduced caregiver reliance, 3) Social integration, 4) Attitudinal shifts, 5) Device-related health issues, 6) Public stigma, 7) Achieved "normalcy", 8) Enhanced mobility, 9) Improved daily functioning, 10) Workplace discrimination | Not reported |
| Katherine Froehlich-Grobe, | 1) 11.5% disabled participants, 2) Equal service use, 3) Equal abnormal rates, 4) Equal follow-up, 5) Lower guideline compliance with disability RetryClaude can make mistakes. Please double-check responses. | 1) Program reaching disabled women at population-equivalent rates, 2) Direct provider payments, 3) Equal service utilization once enrolled | 1) 27% lower breast screening compliance odds with disability, 2) 22% lower cervical screening compliance odds with disability, 3) Higher tobacco use (29.7% vs 14.9%), 4) More breast problems and hysterectomies | Not reported | Not specifically reported |
| Jennifer L. Wong, | 1) 90% saw ≥1 provider within 12 months, 2) average 2.82 providers seen per person, 3) 32% faced unmet accommodation needs, 4) 18.98% delayed care due to costs/barriers, 5) 20.02% skipped medical care/equipment due to cost | 1) cohabitation (67.52% fewer reported barriers), 2) higher physical function scores, 3) higher quality of life scores, 4) respectful providers (93% received), 5) accessible entrances (93% received) | 1) no safe transfer devices (69%), 2) no wheelchair weighing capability (67%), 3) no adjustable exam tables (44%), 4) female gender (more barriers), 5) SCI diagnosis (89.72% more barriers than MD), 6) insurance not covering rehabilitation (45.86%), 7) no nearby therapists (27.82%), 8) inaccessible transportation (26.32%), 9) unaffordable out-of-pocket expenses | 1) prioritizing care types when facing cost barriers, 2) foregoing assistive devices most frequently, 3) encountering non-ADA-compliant facilities, 4) feeling respected despite physical barriers, 5) 10% without any provider contact annually | Not reported |
| Daniel R. Terry, | 1) Poor health concept understanding. 2) GP primary trusted provider. 3) High ER usage (47% vs 14-16.5% general population). 4) Comparable GP visits (82.4%). 5) Minimal specialized service utilization (88.2% never used). | 1) Established GP trust. 2) Technology communication aids. 3) NABS free interpreting. 4) Self-developed coping strategies. 5) Family support. | 1) Limited interpreters statewide. 2) Poor English/health literacy. 3) Interpreter wait times. 4) Social isolation. 5) Limited healthcare system knowledge. 6) Low specialized health awareness. 7) Lower education levels. 8) Lower income status. | 1) Unequal access frustration. 2) Self-taught coping. 3) Multiple communication methods required. 4) Hearing interaction discomfort (46.2%). 5) Deaf community preference (88.2%). 6) Hearing community discrimination (46.2%). 7) Forced resilience development. 8) Technology impact on community cohesion. | Not reported |
| Astha Singhal, | 1) 2× higher edentulism, 2) 2× higher unmet dental needs, 3) 38% lower dental visits, 4) 131% higher medical visits, 5) psychological distress partially mediates outcomes | 1) High medical visit rate (90%), 2) potential for medical visit dental referrals, 3) Medicare coverage | 1) Psychological distress (12.5% vs 2.68%), 2) transportation, 3) escort needs, 4) pain, 5) fatigue, 6) cost, 7) lower private insurance (31.4% vs 62.3%) | 1) Higher edentulism (26.94% vs 6.40%), 2) more unmet needs (19.95% vs 11.07%), 3) fewer dental visits (46.21% vs 61.90%), 4) higher distress (5.16 vs 2.28) | Not reported |
| Dong Wook Shin, | 1)Equal stage distribution at diagnosis,2) higher unknown staging in severe disabilities (18.1% vs 16.2%),3)lower surgery rates in disabled (33.1% vs 38.6%),4) higher ADT use (57.9% vs 55%,5) increased overall mortality and cancer-specific mortality . | 1)Universal coverage with 5% co-payment,2)maximum annual copay ceiling for low-income patients (≈$1000). | 1)insufficient staging workup in severe disability,2) minimal surgery for brain/mental impairments (OR 0.29),3)overuse of ADT versus surgery in severely disabled,4)potential ableism influencing care decisions. | Not reported | Not reported |
| Luz Mairena Semeah, | 1) Decreased hospitalization post-modification. 2) Increased outpatient visits post-modification. 3) Rural-urban difference significant only pre-modification. 4) Urban veterans fewer outpatient visits pre-modification. | 1) Home modifications. 2) Marriage. 3) Preventative care access. 4) Female/Black status post-modification. | 1) Unmarried status. 2) Rural location. 3) Transportation limitations. 4) Skilled worker shortage. 5) Substandard housing. | 1) Financial hardship. 2) Credit problems. 3) Enhanced independence. 4) Improved home safety. 5) Better home-based care access. | Not reported in the study. |
| Dikaios Sakellariou, | Unresponsive healthcare systems 2) Multi-level barriers 3) Three discrimination pathways: normativity expectations, lack of disability awareness, discontinuity of care 4) Symptom misattribution 5) Diagnostic delays 6) Poor specialist coordination | Self-advocacy 2) Responsive professionals 3) Patient-initiated coordination | 1)Non-disabled-centric procedures 2) Limited disability awareness 3) Fragmented specialist care 4) Inaccessible facilities/equipment 5) Symptom misattribution 6) Poor information timing 7) Poverty | 1)Diagnostic delays 2) Treatment complications 3) Inaccessible procedures 4) Need to repeatedly explain/justify needs 5) Compounding effects of multiple challenges 6) Reluctance to characterize care as substandard | 1)Performing medical procedures staff refused 2) Repeated advocacy despite dismissal 3) Witnessing preventable complications 4) Frustration with uncoordinated care 5) Observing missed diagnoses |
| Janet Pearson, | 1) 85.9% eligible had cervical smears (90.2% within 3 years); 2) 92.5% eligible had mammograms (85.4% within 2 years); 3) Only 38.1% self-examined breasts; 4) No screening rate differences across disability types | 1) Family/partner cohabitation; 2) Employment; 3) Sufficient income | 1) Multiple disabilities; 2) Non-English language preference; 3) Insufficient income; 4) Transportation difficulties; 5) Physical accessibility issues; 6) Information format limitations; 7) Lack of knowledgeable providers | 1) Multiple disabilities = more service barriers; 2) Painful examinations; 3) Inaccessible locations; 4) 27.4% untaught on breast self-examination; 5) 7.1% physically unable to self-examine | Not reported |
| Jill Hanass-Hancock | 1) Limited physical accessibility, 2) Absence of reasonable accommodations, 3) Poor healthcare worker training on disability, 4) Weak referral systems, 5) Inadequate service integration | 1) Disability help desks, 2) Preferential treatment in queues, 3) Visual/simplified information, 4) Improved staff attitudes, 5) Physical accessibility enhancements | 1) Inaccessible facilities, 2) No Braille/sign language, 3) Budget constraints, 4) Transportation challenges, 5) Negative attitudes/misconceptions, 6) Poor screening tools | 1) Increased empowerment/advocacy skills, 2) Faced misconceptions about sexuality and HIV risk, 3) Mixed success initiating facility changes, 4) Some experienced staff resistance | Not reported |
| Aviwe S Mgibantaka, | 1) 40% mortality by year 3, 2) lower mortality in rural hospital (16% vs 43% urban), 3) high HbA1c increased mortality risk by 4%, 4) major amputations doubled mortality risk, 5) comorbidities significantly increased mortality. | 1) multidisciplinary referrals (97.5%), 2) longer rural hospital stays (9 vs 6 days), 3) rural patients returned to referring hospitals (83.3%). | 1) poor glycemic control (HbA1c 11.4%), 2) high unemployment (75.2%), 3) limited podiatry services, 4) geographical barriers, 5) limited specialist care, 6) insufficient education programs, 7) poor self-management. | 1) predominance of major amputations (78.1%), 2) previous amputations common (20.2%), 3) comorbidities heightened mortality risk. | Not reported |
| Namkee G Choi, | 1) 9.9% uninsured, 2) 7× higher care postponement, 3) 15-23% provider contact odds, 4) reduced specialist/dental/eye visits, 5) fewer hospitalizations, 6) uninsured trend: 14.5%→7.7%→9.4% | 1) Medicare/Medicaid for disabled/low-income, 2) ACA reduced uninsured rates, 3) safety-net providers, 4) premium subsidies | 1) costs (40% foregone care), 2) no employer insurance, 3) unaffordable premiums, 4) subsidy ineligibility, 5) immigration status, 6) knowledge gaps, 7) limited safety-nets, 8) ACA erosion | 1) functional limitations without insurance = major barriers, 2) underdetected chronic conditions, 3) competing financial priorities (healthcare vs. housing/food) | Not reported |
| Elham Mahmoudi, | 1)Commercial insurance: better preventive care than Medicare. 2) Less prevention: more hospitalizations. 3) Medicare: 1.5-2.3× higher hospitalization risk. 4) Dual-eligibles: worst outcomes. 5) Wellness visits: 20-40% fewer hospitalizations. | 1)Commercial insurance. 2) Medicare Advantage programs. 3) Annual wellness visits. 4) Non-dual eligibility. | 1)Traditional Medicare. 2) Dual eligibility. 3) Black/Hispanic race/ethnicity with Medicare. 4) Low Medicaid reimbursements. 5) High insurance costs. | Not reported | Not reported |
| Andrew Sentoogo Ssemata, | 1) Advocacy needed for healthcare rights. 2) Community-based services reduce travel burden. 3) Disability training for providers essential. 4) Physical accessibility critical. 5) Special clinic days recommended. 6) Inclusion in management committees important. 7) Rehabilitation services needed. | 1) Peer support networks. 2) Medical savings groups. 3) Disabled healthcare workers. 4) Provider-patient dialogue sessions. 5) Management committee representation. 6) Affordable assistive devices. 7) Patient navigation services. | 1) Limited rights awareness. 2) Financial constraints. 3) Long distances. 4) Inaccessible infrastructure. 5) Untrained providers. 6) Stigma. 7) Expensive assistive devices. 8) Limited rehabilitation. | 1) Excluded from decisions. 2) Financial barriers. 3) Inaccessible facilities. 4) Provider knowledge gaps. 5) Limited specialized services. 6) Unaffordable assistive devices. 7) Stigmatization. | Not reported |
| Manjula Marella, | (1) Lower well-being scores, (2) reduced health services access, (3) limited employment participation, (4) restricted rehabilitation services, (5) poor education access, (6) reduced social welfare program access, (7) exclusion from disaster management, (8) decreased social/religious participation | Not reported | (1) Information gaps, (2) costs, (3) disability itself, (4) negative family attitudes, (5) physical inaccessibility, (6) service unavailability, (7) opportunity scarcity for employment, (8) caregiving responsibilities. | (1) Higher psychological distress, (2) reduced well-being, (3) participation restrictions, (4) community stigma, (5) employment/social participation limitations. | Not reported |
| Shikha Gupta, | 1) Non-rationing precedes cuts. 2) Insurance inadequate. 3) Higher costs plus fewer resources equals extreme stress. 4) Cyclical burden affects multiple domains. | 1) Doctors prescribing cheaper alternatives. 2) Employer drug insurance. 3) Public drug benefits. 4) Clinician awareness of barriers. 5) Cost discussions initiation. | 1) Complex paperwork. 2) High deductibles. 3) Formulary restrictions. 4) Approval delays. 5) Limited coverage. 6) Income instability. 7) Poor health increasing needs. 8) No affordable alternatives. 9) Limited doctor communication. | 1) Choosing between medications/basic needs. 2) Medication rationing. 3) Cutting essentials. 4) Symptom worsening. 5) Psychological distress. 6) Asset liquidation/borrowing. 7) Job retention despite health decline. 8) Increased hospitalizations. 9) Stress-scarcity cycle. | Not reported |
| Francesca M Nicosia, | 1) Three measurement aspects: screening, documentation, data use, 2) Function rarely measured routinely, 3) Measurement-care connection critical, 4) Non-standardized data, 5) Difficult tracking over time. | 1) Electronic reminders/templates, 2) Pre-visit screening, 3) Interdisciplinary teams, 4) Provider valuing function, 5) Positive patient relationships, 6) Documentation templates, 7) Workflow integration, 8) Electronic dashboards. | 1) Time constraints, 2) Competing priorities, 3) Understaffing, 4) Non-standardized processes, 5) Unclear roles, 6) Patient underreporting, 7) Patient impairments affecting assessment, 8) Ad-hoc approaches, 9) Cumbersome documentation, 10) No standardized data location, 11) Weak measurement-outcome connection, 12) Limited services, 13) Provider knowledge gaps. | 1) Underreporting due to pride, 2) Self-reporting difficulties with cognitive/sensory impairments, 3) Delayed service access. Experiences of caregivers: 1) Raising functional concerns, 2) Infor | 1) Raising functional concerns, 2) Information source for cognitively impaired patients. |
| Shengxuan Jin, | 1)Decreasing outpatient utilization (23.4% in 2011 to 20.7% in 2018) 2) Increasing inpatient utilization (12.1% in 2011 to 27% in 2018) 3) Increasing self-treatment (55.6% in 2011 to 68.3% in 2018) 4) Pro-rich inequalities across all health services 5) Highest inequality in inpatient services 6) Highest disparities for physical disabilities in inpatient services 7) Highest disparities for intellectual disability in self-treatment | 1)Higher socioeconomic status 2) Having medical insurance (OR=2.115 for inpatient services) 3) Higher education level 4) Social activity participation (OR=1.362 for outpatient services) 5) Child financial support | 1)Economic factors (main contributor to inequality) 2) Low education level 3) Lack of medical insurance 4) Rural residence 5) Poor service accessibility 6) Type of disability affecting differential access 7) Proportion of people who "should have been hospitalized but were not" increased over time | 1)Passive healthcare-seeking behavior 2) High reliance on self-treatment (increasing to 68.3% by 2018) 3) Different utilization patterns by disability type 4) Multiple disabilities had highest outpatient utilization (27.1%) 5) Speech impediments had lowest outpatient utilization (12.0%) 6) Financial burden from rehabilitation costs | Not reported |
| Badriyeh Karami, | 1) 71.84% no hospitalization; 2) males 2.11× higher utilization; 3) Civil Servants insurance 3.44× higher odds; 4) moderate disability 2.13× higher odds; 5) concentration among lower SES; 6) gender 21.92% contribution to inequality | 1) male gender; 2) Civil Servants insurance; 3) moderate disability severity; 4) Military insurance coverage | 1) lower SES; 2) no insurance; 3) limited rehabilitation coverage; 4) no home care coverage; 5) female gender | Not reported | Not reported |
| Jacqueline Moodley | 1) Disabled reported poorer health. 2) Higher TB prevalence (6% vs 3%). 3) Higher chronic disease rates. 4) More healthcare consultations (44% vs 20% in last 30 days). 5) Lower medical insurance coverage (10% vs 18%). | Not reported | 1) Lower income. 2) Limited medical insurance. 3) Dependency on overloaded public healthcare. 4) Transport/geographic/physical accessibility challenges. | 1) Poorer self-reported health. 2) Higher disease burden. 3) Frequent provider consultations. 4) Greater public healthcare reliance. | Not reported in the article |

**Table S3:** *Risk of bias Cross sectional Studies (CASP checlist).*

| **Author_Year** | **Q1** | **Q2** | **Q3** | **Q4** | **Q5** | **Q6** | **Q7** | **Q8** | **Q9** | **Q10** | **Q10** | **Overall Judgment** |
| --- | --- | --- | --- | --- | --- | --- | --- | --- | --- | --- | --- | --- |
| Charlotte Featherstone_2022 (38) | Yes | Yes | No | Yes | Yes | Can’t tell | Yes | Yes | Yes | Yes | Moderate | M |
| Arnold_2024 (51) | Yes | Yes | No | Yes | Yes | Can’t tell | Yes | Yes | Yes | No | High | L |
| Nicole David_2024 (71) | Yes | Yes | No | Yes | Yes | Yes | Yes | Yes | Yes | Yes | High | L |
| Ahmed A. Mohamed_2024 (72  ) | Yes | Yes | No | Yes | Yes | Yes | Yes | Yes | Yes | No | moderate | M |
| Lisa A. Razzano_2014 (14) | Yes | Yes | Can’t tell | Yes | Yes | Can’t tell | Yes | Yes | Yes | Yes | High | L |
| Kevin Lu,_2022(15) | Yes | Yes | Yes | No | Yes | Can’t tell | Yes | Yes | Yes | No | Moderate | M |
| Rafat Hussain_2020 (53) | Yes | Yes | No | Can’t tell | Yes | Can’t tell | Yes | Yes | Yes | No | Moderate | M |
| J.D. Reinhardt_2020 (74) | Yes | Yes | No | Yes | Yes | Can’t tell | Yes | Yes | Yes | No | High | L |
| Dan Qiu_2024 (73) | Yes | Yes | No | Can’t tell | Yes | Yes | Yes | Yes | Yes | No | Moderate | M |
| Monika Mitra_2021 (17) | Yes | Can’t tell | Can’t tell | Yes | Yes | Yes | Yes | Yes | Yes | Yes | High | L |
| Marisa Brown_2016(18) | Yes | Can’t tell | No | No | Can’t tell | Yes | Yes | No | Yes | No | Low | H |
| David Mason_2022 (46) | Yes | Yes | Yes | Yes | Yes | Yes | Yes | Yes | Yes | Yes | High | L |
| Dr Jean-Francois Trani_2017(75) | Yes | Yes | Can’t tell | Yes | Yes | Yes | Yes | Yes | Yes | Can’t tell | High | L |
| Carrie E. Henning-Smith_2016 (19) | Yes | Yes | Yes | Can’t tell | Yes | Yes | Yes | Yes | Yes | Yes | High | L |
| Nancy A. Miller_2014(20) | Yes | Yes | Yes | Can’t tell | Yes | Yes | Yes | Yes | Yes | Can’t tell | High | L |
| Mohammad Hifz Ur Rahman_2019 (68) | Yes | Yes | Yes | Yes | Yes | Can’t tell | Yes | Yes | Yes | Yes | High | L |
| Hussain Zandam,2019  (76) | Yes | Yes | Yes | Yes | Yes | Yes | Yes | Yes | Yes | Can't tell | High | L |
| Sara H Rotenberg,2024(77) | Yes | Yes | Yes | Yes | Yes | No | Yes | Yes | Yes | Can't tell | Moderate | M |
| Claire Ryan, 2018(21) | Yes | Yes | No | Yes | Yes | Can't tell | Yes | Yes | Yes | No | Moderate | M |
| Kavita Makan,2024 (58) | Yes | Yes | No | Yes | Yes | Can't tell | Yes | Yes | Yes | No | Moderate | M |
| Barbara Kołłątaj, 2015 (78) | Yes | Yes | Can't tell | No | Yes | Yes | Yes | Yes | Yes | Can't tell | High | L |
| Olena Bychkovska,2023 (70) | Yes | Yes | No | Yes | Yes | Yes | Yes | Yes | Yes | Can't tell | High | L |
| Niu Tian,2022 (24) | Yes | Yes | Yes | Yes | Yes | Yes | Yes | Yes | Yes | Yes | High | L |
| Amber Willink,2021 (25) | Yes | Yes | Yes | Yes | Yes | Yes | Yes | Yes | Yes | Yes | High | L |
| Sarah E. Bauer,2016(28) | Yes | Yes | Yes | Yes | Yes | Yes | Yes | Yes | Yes | Yes | High | L |
| Kevin J. Bennett, 2016 (29) | Yes | Yes | Yes | Yes | Yes | Yes | Yes | Yes | Yes | Yes | High | L |
| Katherine Froehlich-Grobe, 2016 (31) | Yes | Yes | Yes | Yes | Yes | Yes | Yes | Yes | Yes | Can't tell | High | L |
| Jennifer L. Wong, 2019 (30) | Yes | Yes | Yes | Can't tell | Yes | Yes | Yes | Yes | Yes | Can't tell | Yes | M |
| Astha Singhal, 2016 (32) | Yes | Yes | Yes | Yes | Yes | Yes | Yes | Yes | Yes | Yes | high | L |
| Luz Mairena Semeah, 2021 (33) | Yes | Yes | Yes | Yes | Yes | Yes | Yes | Yes | Yes | Can't tell | high | L |
| Janet Pearson, 2020(79) | Yes | Yes | No | Yes | Yes | Can't tell | Yes | Yes | Yes | Can't tell | Yes | M |
| Namkee G Choi,2020(34) | Yes | Yes | Yes | Yes | Yes | Yes | Yes | Yes | Yes | Yes | High | L |
| Manjula Marella,2016 (80) | Yes | Yes | Yes | Yes | Yes | Yes | Yes | Yes | Yes | No | High | L |
| Badriyeh Karami,2024(81) | Yes | Yes | No | Yes | Can't tell | Can't tell | Yes | Yes | Yes | No | Yes | M |
| Jacqueline Moodley,2015(61) | Yes | Yes | Yes | Can't tell | Yes | Yes | Yes | Yes | Yes | Yes | High | L |
| Shengxuan Jin,2024 (82) | Yes | Yes | Yes | Yes | Yes | Yes | Yes | Yes | Yes | Yes | High | L |

Q1- Did the study address a clearly focused issue?

Q2- Did the authors use an appropriate method to answer their question?

Q3-Were the subjects recruited in an acceptable way?

Q4- Were the measures accurately measured to reduce bias?

Q5-Were the data collected in a way that addressed the research issue?

Q6- Did the study have enough participants to minimise the play of chance?

Q7-How are the results presented and what is the main result?

Q8-Was the data analysis sufficiently rigorous?

Q9- Is there a clear statement of findings?

Q10- Can the results be applied to the local population?

Q10- How valuable is the research?

**Table S4:** *Risk of bias Cohort Studies (CASP checklist)*

| **Author_Year** | **Q1** | **Q2** | **Q3** | **Q4** | **Q5 (a)** | **Q5 (b)** | **Q6 (a)** | **Q6 (b)** | **Q7** | **Q8** | **Q9** | **Q10** | **Q11** | **Q12** | **Overall Judgment** |
| --- | --- | --- | --- | --- | --- | --- | --- | --- | --- | --- | --- | --- | --- | --- | --- |
| Laura Hughes-McCormack_2020 (39) | Yes | Can’t tell | Yes | No | Can’t tell | No | No | Yes | Yes | Yes | Can’t tell | Yes | Yes | Yes | H |
| R.S. Balogh_2015 (63) | Yes | Yes | Yes | Yes | Yes | Yes | Yes | Yes | Yes | Yes | Yes | Yes | Yes | Yes | L |
| Janet Golder_2024 (57) | Yes | Can’t tell | Can’t tell | Yes | No | No | Yes | Yes | Yes | Yes | Yes | Can’t tell | Yes | Yes | M |
| Laura McKernan Ward_2024 (43) | Yes | Yes | Yes | Yes | Can’t tell | Can’t tell | Yes | Yes | Yes | Yes | Yes | Yes | Yes | Yes | M |
| R Asaad Baksh_2021(45) | Yes | Can’t tell | No | Yes | Yes | Yes | Can’t tell | Yes | Yes | Yes | Yes | Can’t tell | Yes | Yes | M |
| Stephanie E Rogers_2015(36) | Yes | Yes | Yes | Yes | Yes | Yes | Yes | Yes | Yes | Can’t tell | Yes | Can’t tell | Yes | Yes | L |
| Ana Oña, 2023(69) | Yes | No | Yes | Yes | Yes | Can'tell | Yes | Yes | Can't tell | Can't tell | Yes | Yes | No | Yes | M |
| Jihyun Kwon, 2019(83) | Yes | Yes | Yes | Yes | Yes | Yes | Yes | Yes | Yes | Yes | Yes | Yes | Yes | Yes | L |
| Fareha Nishat,2021(64) | Yes | Yes | Yes | Yes | Yes | Yes | Yes | Yes | Yes | Yes | Yes | Yes | Yes | Yes | L |
| Seon Mee Park, 2022 (85) | Yes | Yes | Yes | Yes | Yes | Yes | Yes | Yes | Yes | Yes | Yes | Yes | Yes | Yes | L |
| Dong Wook Shin, 2021 (84) | Yes | Yes | Yes | Yes | Yes | Yes | Yes | Yes | Yes | Yes | Yes | Yes | Yes | Yes | L |
| Aviwe S Mgibantaka, 2024(62) | Yes | No | Yes | Yes | Yes | Yes | Yes | No | No | No | No | Yes | no | no | H |
| Elham Mahmoudi,2024(35) | Yes | Yes | Yes | Yes | Yes | Yes | Yes | Yes | Yes | Yes | Yes | Yes | no | Yes | L |
| Carmen E Capo-Lugo,2019(22) | Yes | Yes | Yes | Yes | Yes | Yes | Yes | Yes | Yes | Can't tell | Yes | Yes | no | Yes | L |
| Ali Lakhani,2022(56) | Yes | Yes | Yes | Yes | Yes | Yes | Yes | Yes | Yes | Can't tell | Yes | Yes | no | Yes | L |

Q1-Did the study address a clearly focused issue

Q2-Was the cohort recruited in an acceptable way?

Q3-Was the exposure accurately measured to minimise bias?

Q4-Was the outcome accurately measured to minimise bias?

Q5- a) Have the authors identified all important confounding factors?

Q5- b) Have they taken account of the confounding factors in the design and/or analysis?

Q6-a) Was the follow up of subjects complete enough?

Q6-b) Was the follow up of subjects long enough?

Q7- What are the results of this study?

Q8- How precise are the results?

Q9- Do you believe the results?

Q10- Can the results be applied to the local population?

Q11- Do the results of this study fit with other available evidence?

Q12- What are the implications of this study for practice?

**Table S5:** *Risk of bias Qualitative Studies(CASP checklist).*

| **Author_Year** | **Q1** | **Q2** | **Q3** | **Q4** | **Q5** | **Q6** | **Q7** | **Q8** | **Q9** | **Q10** | **Overall Judgment** |
| --- | --- | --- | --- | --- | --- | --- | --- | --- | --- | --- | --- |
| Avra Selick_2018(67) | Yes | Yes | Yes | Yes | Yes | no | yes | yes | yes | Yes | L |
| Sebastian Dern_2016(86) | Yes | Yes | Can’t tell | Yes | Yes | no | cant tell | Yes | yes | Yes | M |
| Maji Hailemariam_2016(87) | Yes | Yes | Yes | No | Yes | no | yes | yes | yes | Yes | L |
| Melissa L. Desroches_2024(13) | Yes | Yes | Yes | Yes | Yes | cant tell | yes | yes | yes | Yes | L |
| Errol Cocks_2014(52) | Yes | Yes | Yes | Can’t tell | Yes | cant tell | yes | cant tell | cant tell | Yes | M |
| Amy Oliver_2024(40) | Yes | Yes | Yes | Can’t tell | Yes | cant tell | yes | cant tell | cant tell | Yes | M |
| Sarah Wigham_2022(41) | Yes | Yes | Yes | Yes | Yes | cant tell | cant tell | yes | yes | Yes | L |
| Michael Brown_2016(42) | Yes | Yes | Yes | Yes | Yes | cant tell | cant tell | Can’t tell | Yes | Yes | L |
| Natasha Layton_2024(54) | Yes | Yes | Yes | Yes | Can’t tell | Can’t tell | Yes | Can’t tell | Yes | Yes | M |
| Veronika Reichenberger_2024 (88) | Yes | Yes | Yes | Yes | Can’t tell | Can’t tell | Yes | No | No | Yes | M |
| Bougangue Bassoumah,2019 (89) | Yes | Yes | Yes | Yes | Yes | Yes | Yes | Yes | Yes | Yes | L |
| Reuben Kalavina,2019(90) | Yes | Yes | Yes | Yes | Yes | No | Yes | Yes | Yes | Yes | L |
| Robert Kokou Dowou,2023(91) | Yes | Yes | Yes | Yes | Yes | No | Yes | Yes | Yes | Yes | L |
| Rajeswaran Thiagesan,2024(92) | Yes | Yes | Yes | Yes | Yes | Yes | Yes | Yes | Yes | Yes | L |
| Elise‐Marie Dilger, 2024(93) | Yes | Yes | Yes | Yes | Yes | Yes | Yes | Yes | Yes | Yes | L |
| Rosemary B Hughes,2022 (23) | Yes | Yes | Yes | Yes | Yes | Yes | Yes | Yes | Yes | Yes | L |
| Michelle M. Y. Wong,2022(65) | Yes | Yes | Yes | Yes | Yes | No | Yes | Yes | Yes | Yes | L |
| Tare Lowe, 2024(94) | Yes | Yes | Yes | Yes | Yes | Yes | Yes | Yes | Yes | Yes | L |
| Verusia Chetty,2016(59) | Yes | Yes | Yes | Yes | Yes | No | Yes | Yes | Yes | Yes | L |
| Whitney Powell,2019(26) | Yes | Yes | Yes | Yes | Yes | No | Yes | Yes | Yes | Yes | L |
| Mansha Mirza,2020(27) | Yes | Yes | Yes | Yes | Yes | No | Yes | Yes | Yes | Yes | L |
| Ruth Bailey,2019(49) | Yes | Yes | Yes | Yes | Yes | Can't tell | Can't tell | Yes | Yes | Yes | L |
| Neelam Borade,2019(96) | Yes | Yes | Yes | Yes | Yes | No | Yes | Yes | Yes | Yes | L |
| Andrew Sentoogo Ssemata,2024(95) | Yes | Yes | Yes | Yes | Yes | Yes | Yes | Yes | Yes | Yes | L |
| Shikha Gupta,2019(66) | Yes | Yes | Yes | Yes | Yes | Can't tell | Yes | Yes | Yes | Yes | L |
| Francesca M Nicosia,2018(37) | Yes | Yes | Yes | Yes | Yes | Can't tell | Yes | Yes | Yes | Yes | L |
| Dikaios Sakellariou,2019 (48) | Yes | Yes | Yes | Yes | Yes | Can't tell | Yes | Yes | Yes | Yes | L |
| Dikaios Sakellariou,2019(50) | Yes | Yes | Yes | Yes | Yes | Can't tell | Yes | Yes | Yes | Yes | L |

Q1- Was there a clear statement of the aims of the research?

Q2- Is a qualitative methodology appropriate?

Q3- Was the research design appropriate to address the aims of the research?

Q4- Was the recruitment strategy appropriate to the aims of the research?

Q5- Was the data collected in a way that addressed the research issue?

Q6- Has the relationship between researcher and participants been adequately considered?

Q7- Have ethical issues been taken into consideration?

Q8- Was the data analysis sufficiently rigorous?

Q9- Is there a clear statement of findings?

Q10- How valuable is the research?

**Table S6:** *Risk of bias Mixed methods Studies (MMAT checklist).*

| **Author_Year** | **Q1** | **Q2** | **Q3** | **Q4** | **Q5** | **Overall Judgment** |
| --- | --- | --- | --- | --- | --- | --- |
| Stian H Thoresen_2017(97) | Yes | No | No | Yes | Yes | M |
| Fintan Sheerin_2024(98) | Yes | Yes | Yes | Yes | Yes | L |
| Jill Bradshaw_2024(47) | Yes | Can’t tell | Yes | Yes | Yes | L |
| Daniel R. Terry, 2015(55) | Yes | No | No | No | No | M |
| Jill Hanass-Hancock,2014(60) | Yes | Yes | Yes | Yes | Yes | L |

Q1- Is there an adequate rationale for using a mixed methods design to address the research question?

Q2- Is there an adequate rationale for using a mixed methods design to address the research question?

Q3-Is there an adequate rationale for using a mixed methods design to address the research question?

Q4- Are divergences and inconsistencies between quantitative and qualitative results adequately addressed?

Q5-Do the different components of the study adhere to the quality criteria of each tradition of the methods involved?

**Table S7:** *Risk of bias RCT Studies (CASP checklist).*

| **Author_Year** | **Q1** | **Q2** | **Q3** | **Q4 (a)** | **Q4 (b)** | **Q4**  **(c)** | **Q5** | **Q6** | **Q7** | **Q8** | **Q9** | **Q10** | **Q11** | **Overall Judgment** |
| --- | --- | --- | --- | --- | --- | --- | --- | --- | --- | --- | --- | --- | --- | --- |
| E. Sally Rogers_2016(16) | Yes | Yes | Can’t tell | Can’t tell | Can’t tell | Can,t tell | Yes | Yes | Yes | No | Can’t tell | Can’t tell | Can’t tell | M |

Q1- Did the study address a clearly formulated research question?

Q2- Was the assignment of participants to interventions randomised?

Q3- Were all participants who entered the study accounted for at its conclusion?

Q4- a) Were the participants ‘blind’ to intervention they were given?

Q4- b) Were the investigators ‘blind’ to the intervention they were giving to participants?

Q4- c) Were the people assessing/analysing outcome/s 'blinded'?

Q5- Were the study groups similar at the start of the randomised controlled trial?

Q6- Apart from the experimental intervention, did each study group receive the same level

of care (that is, were they treated equally)?

Q7- Were the effects of intervention reported comprehensively?

Q8- Was the precision of the estimate of the intervention or treatment effect reported?

Q9- Do the benefits of the experimental intervention outweigh the harms and costs?

Q10- Can the results be applied to your local population/in your context?

Q11-Would the experimental intervention provide greater value to the people in your care than any of the existing interventions?

**Table S8:** *Risk of bias Case report studies (JBI checklist).*

| **Author_Year** | **Q1** | **Q2** | **Q3** | **Q4** | **Q5** | **Q6** | **Q7** | **Q8** | **Overall Judgment** |
| --- | --- | --- | --- | --- | --- | --- | --- | --- | --- |
| Charlotte Emily  Mott_2019(44) | Yes | Can’t tell | Yes | Yes | Yes | Yes | Can’t tell | Yes | M |

Q1- Were patient’s demographic characteristics clearly described?

Q2- Was the patient’s history clearly described and presented as a timeline?

Q3- Was the current clinical condition of the patient on presentation clearly described?

Q4- Were diagnostic tests or assessment methods and the results clearly described?

Q5- Was the intervention(s) or treatment procedure(s) clearly described?

Q6-Was the post-intervention clinical condition clearly described?

Q7- Were adverse events (harms) or unanticipated events identified and described?

Q8-Does the case report provide takeaway lessons?
